# African regional and national burden of diabetes mellitus and its attributable risk factors from 1990 to 2021: results from the global burden of disease study 2021

**DOI:** 10.3389/fendo.2025.1643999

**Published:** 2025-08-28

**Authors:** Qiu-Yun Wang, Emmanuel Mensah, Zhi-Chao Li, De-Guo Wang, Cui-Wei Zhang, Bruno Miezah Baako, Lei Zha, Xiang Kong

**Affiliations:** ^1^ Department of Gerontology, Geriatric Endocrinology Unit, The First Affiliated Hospital of Wannan Medical College, Wuhu, China; ^2^ Department of Respiratory Medicine, The First Affiliated Hospital of Wannan Medical College, Wuhu, China; ^3^ Department of Internal Medicine, Takoradi Government Hospital, Takoradi, Western Region, Ghana

**Keywords:** diabetes mellitus, disease burden, African region, global burden of disease, public health interventions

## Abstract

**Aims:**

Diabetes mellitus (DM), a chronic disease characterized by hyperglycemia, has become a global health concern. This study evaluates the burden of DM in the African region from 1990–2021 to inform prevention and control strategies.

**Methods:**

Utilizing data from the Global Burden of Disease (GBD) 2021, we analyzed the incidence, prevalence, mortality, disability-adjusted life years, age-standardized rates and estimated annual percentage changes of DM, type 1 DM (T1DM) and type 2 DM (T2DM) in the African region. Decomposition analysis was utilized to explore the contributions of population growth, aging and epidemiological changes to the disease burden. The comparative risk assessment framework was employed to estimate the influence of second-level risk factors on the diabetes burden.

**Results:**

From 1990 to 2021, the disease burden of DM, T1DM and T2DM in the African region increased significantly. The age-standardized incidence rate of DM, T1DM and T2DM increased from 157.284, 5.934 and 151.350 per 100,000 population in 1990 to 250.459, 5.987 and 244.472 per 100,000 population in 2021, respectively. The age-standardized prevalence rate for DM, T1DM and T2DM increased from 2,426.585, 185.366 and 2,241.219 per 100,000 population in 1990 to 4,677.459, 189.384 and 4,488.075 per 100,000 population in 2021. The age-standardized mortality rate for DM and T2DM increased from 39.568 and 38.649 per 100,000 population in 1990 to 44.860 and 44.193 per 100,000 population in 2021. The age-standardized disability-adjusted life years(DALYs)for DM and T2DM increased from 1,123.079 and 1,059.430 per 100,000 population in 1990 to 1,379.240 and 1,329.440 per 100,000 population in 2021. Population growth was identified as the key driver of the increasing disease burden. High body mass index was a major risk factor for DALYs in DM/T2DM. Suboptimal temperature was a major risk factor for DALYs in T1DM. Mauritius had the highest incidence and prevalence rates for DM, T1DM and T2DM, while Kenya and Niger exhibited the lowest rates for DM/T2DM and T1DM, respectively.

**Conclusion:**

Diabetes mellitus (DM) imposes a substantial global disease burden across Africa, necessitating urgent public health interventions to address risk factors and enhance prevention and control measures.

## 1 Introduction

Diabetes mellitus (DM) is a chronic metabolic disorder characterized by hyperglycemia, caused by an absolute or relative deficiency of insulin and impaired glucose utilization. It has become a major global public health issue (1, 2). According to data from the Global Burden of Disease (GBD) 2019, DM is now the eighth leading cause of death and disability worldwide (3). Data from the International Diabetes Federation (IDF) indicate that the global number of DM patients was 463 million in 2019, increased to 537 million in 2021, and is projected to reach 783 million by 2045 (4, 5). These statistics not only reveal the growing prevalence of DM but also highlight the burden it imposes on human health and socio-economic development.

DM can be mainly categorized into two types: type 1 DM (T1DM) and type 2 DM (T2DM). T2DM, the most common subtype of DM, is closely associated with various risk factors, including obesity, an unhealthy diet, genetic predisposition, aging and psychological stress (6, 7). A combination of environmental factors and genetic susceptibility leads to the destruction of pancreatic β-cells, a loss of insulin secretion capacity, and the onset of T1DM, which has far-reaching effects on children and adolescents (8). Although the pathophysiology of DM is well-established, the regional distribution and burden of these types vary significantly across populations, particularly in low and middle-income regions. According to the 2021 IDF Diabetes Atlas, 24 million adults in Africa were estimated to have DM, which is expected to reach 55 million by 2045 (4).

Despite the growing burden, many African health systems remain inadequately equipped to manage diabetes effectively. A major obstacle to addressing diabetes in Africa is the lack of timely, comprehensive, and high-resolution epidemiological data.

Although several studies have examined the global burden of diabetes, few have conducted long-term, country-specific analyses focused on the African context. Existing research is often fragmented, with inconsistent data sources, limited attribution to risk factors, and insufficient tracking of epidemiological trends over time. National-level variations, evolving risk profiles, and the interplay between demographic transitions and disease burden remain inadequately characterized. Furthermore, the high proportion of undiagnosed cases, limited diagnostic infrastructure, and constrained health system capacity in many African countries further obscure a clear understanding of the true burden. This study utilized the GBD 2021 to comprehensively analyze the burden of DM in the African region and its 47 countries from 1990 to 2021. It explored the prevalence trends of DM in the African region and conducted a detailed analysis of multiple indicators, including age-standardized incidence rate (ASIR), age-standardized prevalence rate (ASPR), age-standardized mortality rate (ASMR), age-standardized disability-adjusted life years (ASDR), and major risk factors. The aim of this study is to gain a comprehensive understanding of the current status of DM in the African region and to provide data support and theoretical guidance for the development of effective DM prevention and control policies in the future.

## 2 Methods

### 2.1 Source of data

The data for this study primarily originates from the GBD 2021, which comprehensively covers the global and regional burden information for 371 diseases, injuries, and 88 risk factors across 204 countries and territories from 1990 to 2021. The GBD study provides an in-depth assessment of disease burden across multiple dimensions, including cause, age, gender, year, and location, enabling direct comparisons between different populations, time periods, and regions. These data are accessible through the Global Health Data Exchange (GHDx) platform and can be conveniently accessed and downloaded from the following link: http://ghdx.healthdata.org/gbd-results-tool (9).

### 2.2 Analysis of diabetes disease burden

In the GBD 2021 study, DM is defined as a physician-diagnosed disease identified through diabetes registries or hospital records. This study used the E10 and E11 codes from the International Classification of Diseases, 10th Revision (ICD-10), to define diabetes. To thoroughly analyze the differences in DM across African region and its 47 countries, a regional comparative analysis was conducted. These 47 countries include (in alphabetical order): Algeria, Angola, Benin, Botswana, Burkina Faso, Burundi, Cabo Verde, Cameroon, Central African Republic, Chad, Comoros, Congo, Côte d’Ivoire, Democratic Republic of the Congo, Equatorial Guinea, Eritrea, Eswatini, Ethiopia, Gabon, Gambia, Ghana, Guinea, Guinea-Bissau, Kenya, Lesotho, Liberia, Madagascar, Malawi, Mali, Mauritania, Mauritius, Mozambique, Namibia, Niger, Nigeria, Rwanda, Sao Tome and Principe, Senegal, Seychelles, Sierra Leone, South Africa, South Sudan, Togo, Uganda, United Republic of Tanzania, Zambia, and Zimbabwe. A detailed population-level analysis was conducted across different demographic groups (covering age, gender, and specific subgroups) to explore the distribution characteristics of DM. The data were stratified into 21 age groups (covering all age ranges from <5 years to ≥95 years, in 5-year intervals) for both males and females. To accurately quantify the burden of DM, a range of metrics was used, including ASIR, ASPR, ASMR, and ASDR (10). Age-standardized rates were calculated using the GBD reference population. The reported estimates represent the mean values from 500 draws of the estimation distribution, with 95% uncertainty intervals (UIs) derived as the 2.5th and 97.5th percentiles of these draws (9).

### 2.3 Decomposition analysis

In addition, we utilized the Das Gupta decomposition method to analyze the changes in the burden of DM, T1DM, and T2DM from 1990 to 2021, breaking them down into contributions from aging, population growth, and epidemiological changes. This method enabled us to decompose the overall changes in disease burden into these key factors, providing a clearer understanding of how demographic and epidemiological shifts have shaped trends over time. Decomposition analysis allows for a detailed evaluation of the independent contribution of each factor to the overall changes in disease burden. By dissecting these trends, we gained a clearer understanding of the potential drivers of changes in the burden of disease for DM, T1DM, and T2DM in the African region (11).

### 2.4 Attributable risk analysis

This study used the Global Health Data Exchange query tool (https://vizhub.healthdata.org/gbd-results/) to collect data on Level 2 risk factors related to DM. These risk factors include high fasting plasma glucose, high body mass index (BMI), dietary risks, air pollution, low physical activity, tobacco, suboptimal temperature, and alcohol use. Since high fasting glucose was identified as a risk factor for diabetes in GBD 2021 with a PAF of 100%, it was excluded from the analysis. GBD 2021 utilized a comparative risk assessment framework to quantify the relationship between each of the level 2 risk factor and DM DALYs, systematically synthesizing the data through meta-analysis. DisMod-MR 2.1 or spatiotemporal Gaussian process regression was employed to estimate the exposure distribution for each risk factor, and further determine the theoretical minimum risk exposure level. Subsequently, the proportion of disease burden attributable to risk factors was calculated by multiplying DALYs by the population attributable fraction (PAF). The sum of the PAFs may exceed 100% because one or more of these risk factors is partially or wholly mediated through another risk factor or risk factors (12).

### 2.5 Statistical methods

EAPC Model: The estimated annual percentage change (EAPC) and its 95% confidence interval (CI) were calculated using log-linear regression, as follows:


Ln(ASR)=α+βx+ϵ

where x represents the year, and β is the regression coefficient. EAPC is determined as 100[exp(β) - 1], and the 95% CI of EAPC is derived from the standard error produced by the log-linear regression. The interpretation of these results is as follows: If the EAPC estimate and the lower limit of its 95% CI are both greater than 0, the ASR is considered to have an increasing trend. If the EAPC estimate and the upper limit of its 95% CI are both less than 0, the ASR is considered to have a decreasing trend. Otherwise, the ASR is considered stable over time (13).

PAF: Population Attributable Fraction (PAF) is used to quantify the contribution of a specific risk factor to the disease burden. It represents the proportion of the disease burden that could theoretically be avoided if the risk factor were eliminated. PAF is calculated using the following formula:PAF = (RR - 1)/RR,where RR is the relative risk, which indicates the increased risk of developing diabetes for individuals exposed to a specific risk factor compared to those unexposed (12). All statistical analyses and visualizations were conducted using the R statistical software program (version 4.4.2) and JD_GBDR (V2.36, JD Health Technology Co., Ltd). A P value <0.05 was considered statistically significant.

### 2.6 Patient and public involvement

The GBD Study is a collaborative scientific effort that allows for the comparison and replication of the impact of different health conditions at specific points in time. Our study utilized secondary data from this collaborative work, and we did not have direct contact with participants. Patients were not involved in setting the research questions or outcome measures, nor were they involved in the design and implementation of the study.

## 3 Results

### 3.1 Analysis of the diabetes disease burden in the African region

In 2021, the disease burden of DM in the African region remained substantial, with 1,855,560.521 new cases of DM (T1DM: 80,308.093; T2DM: 1,775,252.428), and a total of 30,438,743.994 prevalent cases (T1DM: 1,920,499.635; T2DM: 28, 518, 244.359). The ASIR increased from 157.284 per 100,000 population in 1990 to 250.459 per 100,000 population in 2021 [EAPC: 1.533, 95% CI: 1.518 to 1.549] and ASPR increased from 2426.585 per 100,000 in 1990 to 4677.459 per 100,000 in 2021 [EAPC: 2.148; 95% CI: 2.134 to 2.161]. The estimated number of deaths due to DM was 195,797.614 (T1DM: 6894.506; T2DM: 188903.109). The ASMR increased from 39.568 per 100,000 population in 1990 to 44.860 per 100,000 population in 2021 [EAPC: 0.434, 95% CI: 0.320 to 0.549].The DALYs for DM in 2021 totaled 7,790,380.574 (T1DM: 566194.334; T2DM: 7224186.240).The ASDR increased from 1123.079 per 100,000 population in 1990 to 1379.240 per 100,000 population in 2021 [EAPC: 0.667, 95% CI: 0.596 to 0.738](**Table 1**).

**Table 1:** Number and age-standardized rates per year of incident and prevalent diabetes, deaths from diabetes and DALYs due to diabetes in 1990 and 2021, and estimated annual percentage changes (EAPC) in Africa region for 1990–2021, by types of diabetes.

Types	1990		2021		EAPC 95%CI
	Number (95%UI)	ASR (95%UI)	Number (95%UI)	ASR (95%UI)	
Incidence (95%UI)					
Diabetes mellitus	457642.392 (425122.944,493162.975)	157.284 (145.844,169.800)	1855560.521 (1729656.434,1990120.779)	250.459 (233.235,269.278)	1.533 (1.518,1.549)
Type 1 diabetes	38243.995 (33367.745,44241.239)	5.934 (5.258,6.708)	80308.093 (69265.765,94360.356)	5.987 (5.243,6.852)	0.008 (-0.007,0.023)
Type 2 diabetes	419398.397 (387309.194,453679.322)	151.350 (140.274,164.149)	1775252.428 (1649272.938,1908986.233)	244.472 (227.589,263.158)	1.581 (1.564,1.597)
Prevalence(95%UI)					
Diabetes mellitus	6610505.269 (6083934.409,7154171.872)	2426.585 (2238.805,2630.448)	30438743.994 (28257014.289,32761757.733)	4677.459 (4349.143,5019.363)	2.148 (2.134,2.161)
Type 1 diabetes	810182.185 (694576.801,947842.843)	185.366 (159.050,216.693)	1920499.635 (1618843.423,2279466.056)	189.384 (161.771,224.546)	0.081 (0.067,0.096)
Type 2 diabetes	5800323.084 (5277490.873,6342567.697)	2241.219 (2047.949,2446.427)	28518244.359 (26312848.082,30846511.456)	4488.075 (4168.935,4826.835)	2.271 (2.255,2.287)
Deaths(95%UI)					
Diabetes mellitus	79301.493 (72526.964,87375.583)	39.568 (36.125,43.570)	195797.614 (177737.589,214795.841)	44.860 (41.120,48.862)	0.434 (0.320,0.549)
Type 1 diabetes	4364.814 (3616.749,5237.824)	0.919 (0.719,1.119)	6894.506 (5492.204,8361.955)	0.667 (0.521,0.817)	-0.972 (-1.017,-0.927)
Type 2 diabetes	74936.679 (68635.771,82778.471)	38.649 (35.247-42.592)	188903.109 (171869.474,207348.653)	44.193 (40.466,48.142)	0.461 (0.344,0.578)
Dalys(95%UI)					
Diabetes mellitus	2809702.773 (2544109.455,3121876.871)	1123.079 (1022.598,1244.118)	7790380.574 (6886123.871,9005620.116)	1379.240 (1231.455,1584.652)	0.667 (0.596,0.738)
Type 1 diabetes	345532.334 (270244.329,418062.895)	63.650 (53.469,76.060)	566194.334 (457375.590,679339.680)	49.800 (40.774,59.175)	-0.739 (-0.773,-0.704)
Type 2 diabetes	2464170.439 (2232571.883,2744180.381)	1059.430 (964.363,1173.849)	7224186.240 (6402595.529,8398515.992)	1329.440 (1188.053,1524.459)	0.734 (0.658,0.810)

### 3.2 Decomposition analysis

**Figure 1:**
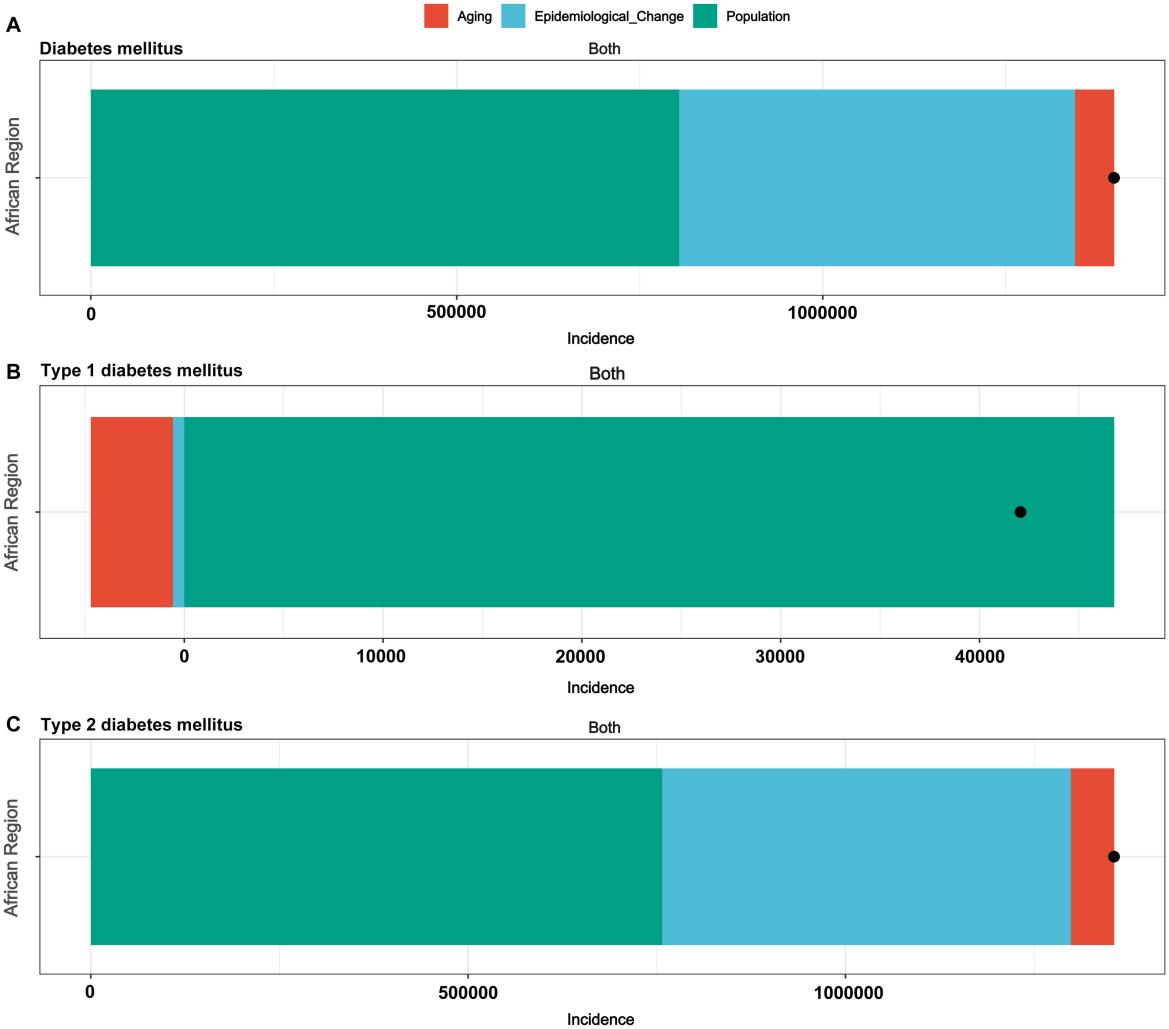
Changes in incidence of diabetes according to aging, population growth and epidemiological change from 1990 to 2021 in the African region. **(A)** Diabetes mellitus, **(B)** Type 1 diabetes mellitus, **(C)** Type 2 diabetes mellitus.

The decomposition analysis was used to assess the impact of aging, population growth and epidemiological changes on the burden of DM, T1DM and T2DM in the African region from 1990 to 2021. The contributions of population growth, aging and epidemiological changes to the increase in the burden of DM were 57.51%, 3.81% and 38.68%, respectively. For the increase in T1DM burden, the contributions were 111.21%, -18.4% and 7.18%, respectively. For the increase in T2DM burden, the contributions were 55.86%, 4.21% and 39.94%, respectively (**Figure 1**, **Table 2**).

**Table 2:** Changes in incidence number of diabetes mellitus according to disease categories from 1990 to 2021.

Location name	Disease	Overall difference	Aging	Population	Epidemiological Change	Aging Percentage	Population Percentage	Epidemiological Change Percentage
African Region	Diabetes mellitus	1397918.13	53268.43	803998.82	540650.87	3.81	57.51	38.68
	Type 1 diabetes	42064.1	-7739.06	46781.51	3021.65	-18.4	111.21	7.18
	Type 2 diabetes	1355854.03	57050.71	757338.48	541464.84	4.21	55.86	39.94

### 3.3 The burden of DM by age and sex

**Figure 2:**
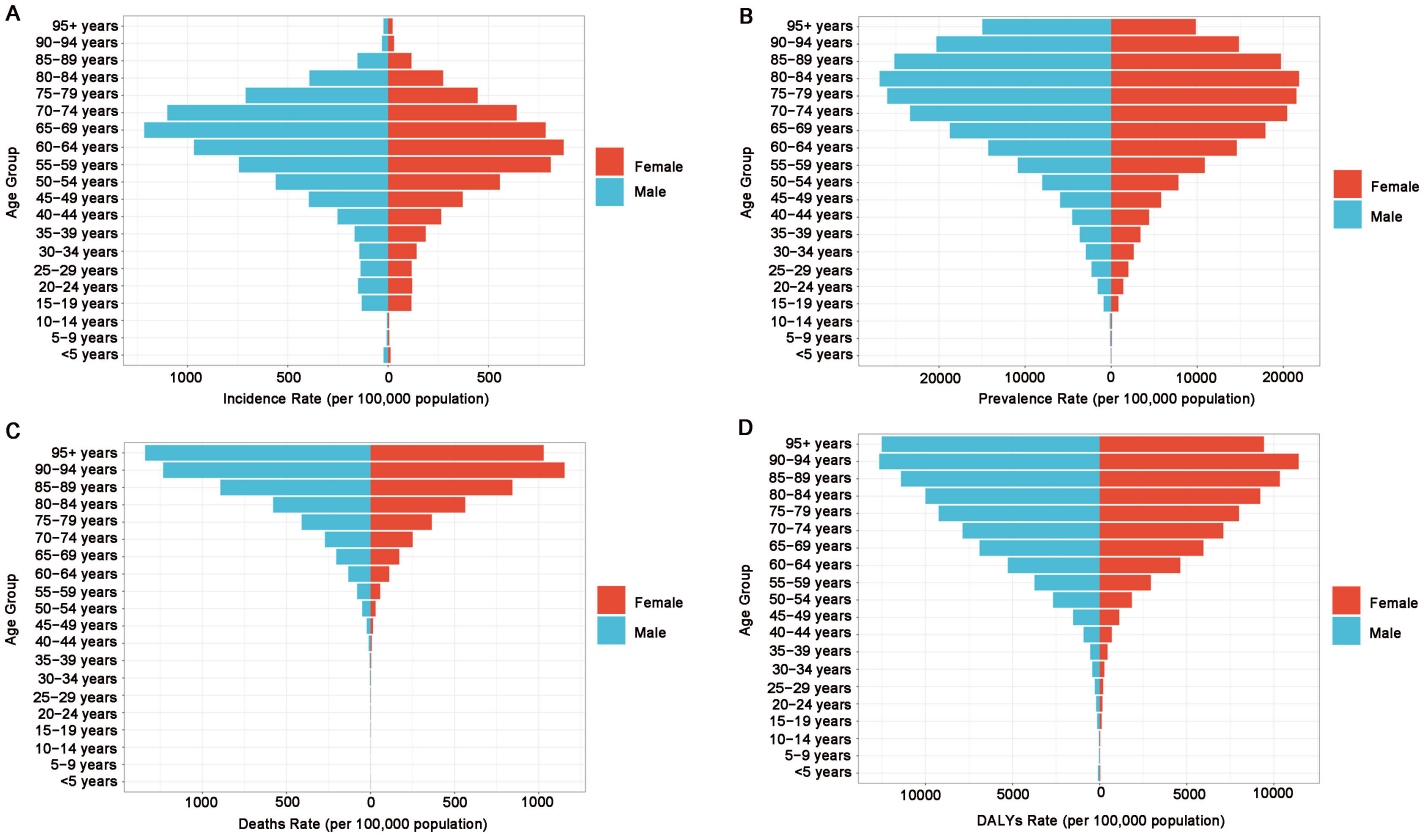
Sex- and age-structured analysis of diabetes mellitus disease burden in 2021. **(A)** Age- and sex-specific incidence rates, **(B)** age- and sex-specific prevalence rates, **(C)** age- and sex-specific death rates, **(D)** age- and sex-specific DALY rates.

In 2021, the burden of DM and T2DM in the African region showed the same trends when stratified by age and sex. The age-standardized incidence and prevalence rates of DM and T2DM increased with age. The ASIR peaked for males in the 65–69 age group and for females in the 60–64 age group. After the age of 59, the ASIR was higher in males than in females. The ASPR for both males and females peaked in the 80–84 age group, and after the age of 64, the ASPR was higher in males than in females (**Figures 2A, B**; **Supplementary Figures S1A, B**). The ASMR peaked in males in the ≥95 age group and in females in the 90–94 age group, in the vast majority of age groups, the ASMR for males is higher than that for females (**Figure 2C**; **Supplementary Figure S1C**). The ASDR for both males and females peaked in the 90–94 age group, in the vast majority of age groups, the ASDR for males is higher than that for females (**Figure 2D**; **Supplementary Figure S1D**).

In 2021, the burden of disease for T1DM by age and sex in the African region has a clear trend of change. The ASIR for both males and females peaked in the < 5 age group. However, after the age of 59, the ASIR was higher in females than in males (**Supplementary Figure S2A**). The ASPR of T1DM began to rise from the < 5 age group, peaking for males in the 55–59 age group and for females in the 60–64 age group. Throughout all age groups, the ASPR was consistently higher in males than in females (**Supplementary Figure S2B**). The ASMR for both males and females peaked in the > 95 age group, and except for the 75–79 age group, the ASMR was consistently higher in males than in females (**Supplementary Figure S2C**). The ASDR for both males and females peaked in the 40–44 age group, with the ASDR consistently higher in males than in females throughout (**Supplementary Figure S2D**).

### 3.4 DM-related DALYs attributed to risk factors

**Figure 3:**
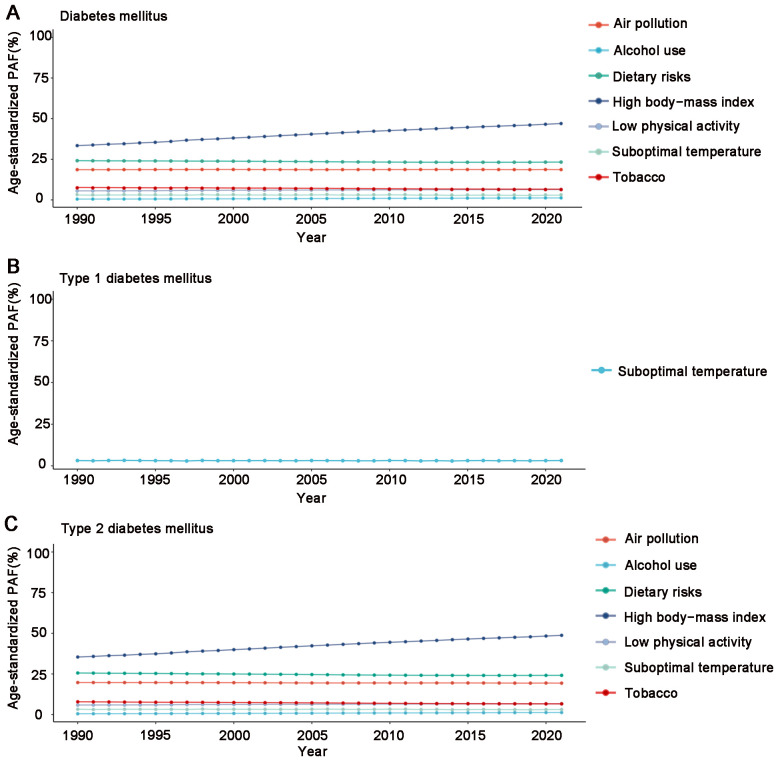
Age-standardized population-attributable fractions (PAF) of disability-adjusted life years (DALYs) of risk factors in the African region during 1990 and 2021. **(A)** Diabetes mellitus, **(B)** Type 1 diabetes mellitus, **(C)** Type 2 diabetes mellitus.

In Africa, the proportion of DALYs caused by individual risk factors for DM, T1DM and T2DM differs. High BMI (46.98%) accounted for the largest proportion of DALYs across all risk factors, indicating its substantial role in DM burden, followed by dietary risks (23.36%), air pollution (18.63%), low physical activity (6.38%), tobacco (6.38%), suboptimal temperature (3.15%) and alcohol use (1.23%). The DALYs rate for T1DM is mainly attributed to suboptimal temperature (3.21%). The DALYs rate for T2DM is mainly attributed to high BMI (48.74%) and dietary risks (24.14%), followed by air pollution (19.33%), low physical activity (6.62%), tobacco (6.61%), suboptimal temperature (3.15%) and alcohol use (1.27%). Notably, the PAF value of high BMI significantly increased from 35.41% in 1990 to 48.74% in 2021 (**Figure 3**; **Supplementary Figure S3**).

### 3.5 National level analysis

Analyzing the 2021 data for diabetes-related ASIR and ASPR, we observed consistency among the countries most severely affected. Mauritius, Kenya and Niger stood out in both metrics. Mauritius recorded the highest ASIR (DM: 641.505, 95% UI: 603.715 to 684.313; T1DM: 12.058, 95% UI: 10.953 to 13.310; T2DM: 629.446, 95% UI: 591.110 to 672.285) and ASPR (DM: 11,198.412, 95% UI: 10,450.807 to 11,952.101; T1DM: 329.140, 95% UI: 279.370 to 381.621; T2DM: 10,869.271, 95% UI: 10,113.975 to 11,631.117) per 100,000 population. Kenya showed the lowest DM/T2DM ASIR (DM: 140.250, 95% UI: 129.413 to 153.285; T2DM: 134.555, 95% UI: 123.503 to 147.532) and ASPR (DM: 2,044.356, 95% UI: 1,872.372 to 2,231.736; T2DM: 1,858.492, 95% UI: 1,674.312 to 2,051.779), while Niger had the lowest T1DM ASIR (4.277, 95% UI: 3.682 to 4.985) and ASPR (128.399, 95% UI: 107.290 to 154.304) (**Table 3**; **Supplementary Tables S1**–**S5**).

**Table 3:** Incidence cases and rates of diabetes mellitus in African countries and estimated annual percentage changes in 1990 and 2021, along with the rankings of African countries based on age-standardized incidence rates in 1990 and 2021.

Incident from diabetes (1990-2021) in Africa							
Location	1990			2021			EAPC 95%CI
	Number (95%UI)	ASR (95%UI)	ASR Ranking	Number (95%UI)	ASR (95%UI)	ASR Ranking	
Algeria	34332.982 (31256.044-37582.642)	212.593 (191.972-236.496)	7	212641.101 (195696.362-229600.217)	484.920 (449.433-522.964)	3	2.727 (2.697-2.758)
Angola	11348.809 (10409.669-12436.640)	200.386 (184.869-217.693)	10	62683.464 (57468.216-68726.209)	306.578 (280.942-332.791)	14	1.442 (1.406-1.477)
Benin	4461.201 (4087.687-4872.140)	170.365 (154.556-185.867)	18	26525.966 (24275.990-29109.896)	312.095 (286.217-342.637)	13	1.873 (1.782-1.963)
Botswana	1275.305 (1173.623-1383.907)	183.013 (168.989-197.039)	12	5837.690 (5434.822-6255.291)	303.646 (285.113-322.420)	15	1.731 (1.692-1.770)
Burkina Faso	7239.156 (6756.368-7791.773)	138.126 (129.188-147.579)	36	31632.176 (29116.542-34086.084)	232.553 (214.144-248.977)	32	1.701 (1.608-1.794)
Burundi	4290.633 (4045.670-4575.540)	139.675 (130.809-149.639)	35	13211.357 (12265.441-14135.953)	175.912 (162.718-188.720)	41	0.679 (0.648-0.710)
Cabo Verde	387.851 (353.773-423.182)	156.831 (143.370-171.369)	23	1797.724 (1644.676-1962.267)	336.160 (307.926-365.731)	11	2.696 (2.583-2.809)
Cameroon	8967.903 (8254.469-9697.577)	154.352 (143.618-166.355)	24	52573.572 (48875.457-56818.481)	264.090 (246.425-283.615)	23	1.830 (1.745-1.915)
Central African Republic	3575.740 (3285.482-3892.061)	219.080 (201.731-239.524)	4	13498.502 (12336.367-14654.642)	341.399 (313.424-369.125)	9	1.474 (1.433-1.514)
Chad	4953.781 (4578.157-5371.103)	140.614 (128.972-153.154)	34	23479.286 (21415.783-25547.272)	246.626 (224.980-267.886)	28	1.797 (1.653-1.941)
Comoros	418.167 (388.842-453.333)	166.478 (154.994-181.148)	19	1475.296 (1369.854-1593.727)	242.781 (225.938-262.553)	31	1.244 (1.216-1.273)
Congo	2663.883 (2459.247-2866.993)	191.033 (178.507-203.145)	11	12409.076 (11413.079-13539.334)	301.023 (279.984-324.102)	16	1.535 (1.493-1.577)
Côte d’Ivoire	10272.671 (9366.061-11193.259)	164.378 (151.525-177.622)	20	50555.737 (46203.211-55050.330)	279.724 (256.991-306.655)	19	1.761 (1.714-1.809)
Democratic Republic of the Congo	33826.833 (31234.810-36610.675)	161.487 (149.446-174.057)	21	145780.209 (133012.596-158790.330)	244.123 (223.728-263.257)	29	1.360 (1.311-1.409)
Equatorial Guinea	452.800 (419.138-490.360)	180.879 (168.534-195.435)	14	3139.605 (2871.509-3433.286)	336.916 (313.748-361.761)	10	2.244 (2.157-2.331)
Eritrea	2569.168 (2372.426-2796.202)	153.042 (141.426-165.362)	25	10089.911 (9304.376-10884.473)	228.115 (211.381-244.866)	34	1.308 (1.287-1.329)
Eswatini	869.443 (810.027-936.165)	234.433 (219.668-251.682)	2	3107.392 (2909.347-3320.450)	417.815 (392.587-442.797)	4	2.027 (1.922-2.133)
Ethiopia	48236.417 (44349.394-52503.314)	177.897 (163.394-193.665)	17	127084.493 (116463.770-138305.751)	194.303 (177.976-212.234)	37	0.143 (0.069-0.217)
Gabon	1432.803 (1330.941-1548.691)	214.883 (200.276-230.805)	5	5240.284 (4873.794-5611.393)	364.943 (341.437-389.010)	7	1.802 (1.734-1.870)
Gambia	737.022 (673.268-798.033)	143.313 (130.859-156.350)	32	3978.362 (3658.060-4336.949)	266.114 (244.249-291.311)	22	2.093 (2.052-2.134)
Ghana	12922.667 (11868.342-14056.437)	148.121 (136.652-160.665)	27	66840.711 (60956.912-72294.620)	271.054 (248.817-294.736)	20	1.980 (1.808-2.152)
Guinea	5323.908 (4932.992-5764.679)	136.860 (127.114-148.742)	37	18913.007 (17533.960-20488.181)	230.302 (212.760-248.453)	33	1.539 (1.458-1.621)
Guinea-Bissau	1011.199 (925.652-1096.284)	180.306 (165.579-195.185)	15	3804.373 (3498.493-4137.962)	299.230 (276.375-323.767)	17	1.668 (1.637-1.699)
Kenya	11833.795 (10925.584-12773.466)	107.532 (99.156-117.454)	47	42932.891 (39496.077-46568.889)	140.250 (129.413-153.285)	47	0.825 (0.763-0.888)
Lesotho	1434.759 (1338.740-1534.831)	149.488 (140.227-159.971)	26	4349.770 (4087.980-4611.387)	324.781 (306.459-342.852)	12	2.789 (2.706-2.872)
Liberia	2324.085 (2135.584-2528.317)	158.484 (145.553-172.676)	22	10327.398 (9378.542-11279.953)	283.508 (258.832-308.231)	18	1.979 (1.942-2.016)
Madagascar	7608.196 (7069.923-8200.191)	116.051 (108.169-125.640)	45	27982.788 (25712.285-30143.723)	161.120 (148.992-173.593)	44	1.046 (1.030-1.063)
Malawi	6025.806 (5665.296-6413.065)	121.429 (114.843-129.148)	43	15599.319 (14716.958-16710.317)	149.033 (140.017-158.400)	46	0.600 (0.572-0.628)
Mali	12029.805 (10912.240-13195.402)	229.748 (208.353-251.433)	3	58790.860 (53771.215-65026.698)	409.899 (375.350-448.881)	5	1.931 (1.861-2.001)
Mauritania	1658.189 (1541.995-1780.260)	134.198 (125.227-144.374)	39	5447.522 (5158.874-5722.824)	189.097 (179.032-197.782)	39	1.062 (0.928-1.196)
Mauritius	2846.348 (2669.567-3047.545)	317.750 (297.175-340.864)	1	11521.648 (10786.373-12327.447)	641.505 (603.715-684.313)	1	2.325 (2.180-2.470)
Mozambique	9223.258 (8677.355-9797.215)	126.332 (119.695-134.268)	41	34640.189 (32010.640-37324.536)	201.171 (186.830-214.169)	36	1.602 (1.555-1.649)
Namibia	1419.221 (1316.977-1535.208)	181.155 (168.536-195.002)	13	4457.984 (4084.685-4852.327)	249.789 (230.085-270.632)	27	1.042 (1.023-1.061)
Niger	5915.209 (5385.293-6435.383)	145.362 (132.817-157.779)	30	32624.421 (29674.370-35834.421)	243.050 (221.464-265.300)	30	1.686 (1.671-1.702)
Nigeria	77940.186 (70958.056-85190.198)	141.602 (128.968-155.684)	33	282106.653 (257956.598-307098.342)	208.791 (191.436-227.485)	35	1.226 (1.186-1.265)
Rwanda	5005.142 (4678.318-5326.966)	135.919 (127.909-143.644)	38	13000.821 (12070.233-13980.298)	153.805 (143.213-164.066)	45	0.261 (0.179-0.343)
Sao Tome and Principe	112.551 (102.726-122.864)	145.652 (131.789-159.791)	29	448.404 (408.474-494.098)	268.454 (243.343-295.278)	21	2.001 (1.987-2.014)
Senegal	8739.127 (7983.611-9517.446)	203.948 (184.527-222.230)	8	39270.758 (36662.414-42046.342)	367.173 (342.196-396.706)	6	2.048 (1.924-2.173)
Seychelles	126.468 (116.413-138.477)	212.816 (194.104-233.781)	6	758.672 (698.141-822.760)	593.293 (550.410-640.444)	2	3.383 (3.214-3.553)
Sierra Leone	3721.449 (3413.874-4059.937)	143.803 (130.940-156.772)	31	14792.493 (13483.500-16079.053)	253.197 (232.194-276.033)	25	1.894 (1.857-1.931)
South Africa	50418.329 (46069.194-55380.895)	201.166 (183.838-221.140)	9	185774.429 (170951.129-202458.029)	346.534 (318.927-376.339)	8	1.901 (1.847-1.955)
South Sudan	4084.862 (3811.081-4408.516)	124.794 (116.758-135.016)	42	9721.500 (8980.457-10497.660)	173.647 (161.445-186.103)	42	1.052 (1.036-1.068)
Togo	2209.554 (2024.683-2392.868)	120.814 (112.044-130.651)	44	11027.549 (10209.584-11827.117)	193.841 (179.073-208.177)	38	1.545 (1.530-1.559)
Uganda	10916.680 (10238.355-11687.167)	127.699 (119.125-137.081)	40	40598.594 (37763.074-43440.722)	181.538 (168.960-194.098)	40	1.122 (1.085-1.159)
United Republic of Tanzania	15711.383 (14792.107-16674.208)	112.745 (106.639-119.114)	46	59022.863 (55303.640-63011.983)	169.651 (158.928-179.960)	43	1.347 (1.306-1.389)
Zambia	7062.207 (6542.277-7583.671)	178.907 (166.187-193.765)	16	29355.904 (27117.828-31871.918)	251.625 (233.219-271.769)	26	1.083 (1.064-1.103)
Zimbabwe	7705.441 (7116.657-8315.817)	147.584 (137.403-160.471)	28	24707.796 (22955.111-26639.519)	254.386 (236.631-273.465)	24	1.933 (1.885-1.981)

Eswatini reported the highest DM/T2DM ASMR (DM: 119.678, 95% UI: 90.920 to 154.886; T2DM: 119.093, 95% UI: 90.487 to 154.287), while Mauritius showed the highest T1DM ASMR (2.519, 95% UI: 2.303 to 2.673). São Tomé and Príncipe had lowest DM/T2DM ASMR (DM: 18.430, 95% UI: 15.613 to 21.671; T2DM: 17.912, 95% UI: 15.261 to 20.980), while Algeria showed the lowest T1DM ASMR (0.317, 95% UI: 0.232 to 0.460) (**Supplementary Tables S6**-**S8**).

Mauritius had the highest ASDR (DM: 3,514.449, 95% UI: 3,183.147 to 3,949.590; T1DM: 128.500, 95% UI: 115.666 to 140.083; T2DM: 3,385.949, 95% UI: 3,066.400 to 3,810.035), with São Tomé and Príncipe had the lowest ASDR (DM: 910.366, 95% UI: 735.778 to 1,149.820; T2DM: 875.708, 95% UI: 705.587 to 1,112.047),while Cape Verde recording the lowest T1DM ASDR (26.749, 95% UI: 20.635 to 34.151) (**Supplementary Tables S9**–**S11**).

## 4 Discussion

This study provides a comprehensive analysis of the burden of diabetes and its attributable risk factors in the African region from 1990 to 2021, based on data from the Global Burden of Disease Study 2021. We found a marked increase in the overall burden of DM, with significant rises in the number of new cases, prevalence, deaths and DALYs, particularly for T2DM. Population growth was identified as the main contributor to this rise. High body mass index was a major risk factor for DALYs in DM/T2DM. Suboptimal temperature was a major risk factor for DALYs in T1DM. Substantial variation was observed across countries, with Mauritius recording the highest incidence and prevalence rates of DM, while Kenya and Niger had the lowest for T2DM and T1DM, respectively.

Our findings indicate a substantial burden of DM in Africa. likely linked to lifestyle changes driven by rapid population growth and economic development. As a region facing widespread poverty and low GDP per capita, Africa allocates only about 2% of its resources to health expenditure-predominantly directed toward infectious diseases such as HIV/AIDS and malaria. Consequently, DM receives limited funding: Africa’s expenditure on DM stands at merely $12.6 billion, accounting for just 1.3% of the global total (14, 15). The scarcity of medical resources contributes to the aggravation of the disease burden. In response to this challenge, African countries need to proactively adjust their public health strategies: Beyond infectious disease control, greater emphasis must be placed on DM prevention and early intervention.

Furthermore, population growth stands as the dominant driver behind the escalating burden of DM, including T1DM and T2DM. This finding aligns with global research outcomes (16). Therefore, future public health initiatives must take population growth into account while implementing targeted and comprehensive prevention and control interventions.

In the African region, ASIR peaks for DM/T2DM occur in the 65–69 age group (males) and 60–64 age group (females), while the ASPR peak in the 80–84 age group, potentially reflecting age-related decline in pancreatic β-cell function and insulin sensitivity (17, 18). The ASMR peaks the age groups of ≥ 95 years (males) and 90–94 years (females), with the ASDR peaking in the 90–94 age group for both genders. These patterns may stem from localized population aging, cumulative diabetes effects, prolonged patient survival, and selection bias, and limitations in data collection (19–21). Women show lower ASMR and ASDR than men, possibly due to women exhibiting a more proactive attitude in healthcare utilization, disease management, and adherence to treatment protocols (22). Notably, African women exhibit higher ASPR than men before age 64 - contrasting global patterns - potentially linked to higher birth rates, which increase the risk of gestational DM (23).

In the African region, the ASIR of T1DM is highest in the < 5 age group, as T1DM is an autoimmune disease closely associated with genetic and environmental factors (such as viral infections), which commonly manifests in childhood. The ASPR for males and females peaked in the age groups of 55–59 and 60–64 years, respectively. This may be attributed to improved insulin accessibility, which increases the survival rate of T1DM patients, as well as advancements in diagnostic techniques leading to higher detection rates of late-onset T1DM (24, 25).

DALYs caused by DM in Africa are influenced by multiple risk factors. Suboptimal temperature is a common risk factor for both T1DM and T2DM, consistent with findings from studies in China (26). Suboptimal temperature is positively associated with diabetes incidence and mortality and has a significant effect on insulin sensitivity (27, 28). For T2DM, additional risk factors include high BMI, dietary risks, air pollution, low physical activity, tobacco and alcohol use. Among them, high BMI is the primary risk factor driving T2DM incidence in the African region. Obesity-induced insulin resistance and metabolic abnormalities are key mechanisms in the development of T2DM. Therefore, weight control and reducing BMI are essential for alleviating the burden of T2DM (29, 30). Dietary risks are another important factor contributing to T2DM development. Unhealthy dietary habits, such as high sugar, high fat and low fiber diets, significantly increase the risk of diabetes (31, 32). Recent studies have shown a significant positive association between air pollution and T2DM risk (33). In particular, fine particulate matter (PM2.5), as a major environmental pollutant, may promote the progression of T2DM by inducing oxidative stress, damaging pancreatic β-cells and reducing insulin synthesis and secretion (34). In addition, low physical activity has been shown to significantly influence T2DM, particularly in low Sociodemographic Index regions, making the promotion of physical activity crucial to reducing this burden (35). Nicotine affects body composition, insulin sensitivity and pancreatic β-cell function, thereby increasing the risk of diabetes (36). Alcohol consumption leads to islet β-cell dysfunction (37).

We conducted an in-depth analysis of the disease burden in 47 African countries and found that Mauritius ranked highest in age-standardized incidence and prevalence rates of DM, T1DM and T2DM. The high ASPR in Mauritius may be associated with rapid urbanization and unhealthy lifestyles. In addition, the higher proportion of South Asian populations in Mauritius, who have a genetic predisposition to diabetes, may further exacerbate the DM burden (38, 39). In contrast, Kenya had the lowest age-standardized incidence and prevalence rates of DM and T2DM, which may be attributed to the country’s relatively low level of urbanization, more traditional dietary habits and higher levels of physical activity. Niger recorded the lowest age-standardized incidence and prevalence rates of T1DM, possibly due to a lower prevalence of HLA variants strongly associated with T1DM, thereby reducing the risk of autoimmune attacks on pancreatic islet cells (40).

This study has several limitations. First, significant differences exist in health information systems and reporting mechanisms across African countries, particularly in low- and middle-income regions and conflict-affected areas. Such disparities may result in localized data gaps, thereby widening the 95% UIs and consequently compromising the precision of burden estimates. Second, this study relies on GBD data, which are subject to a certain degree of lag. This makes it challenging to capture the latest developments in diabetes in the African regions in a timely manner. Lastly, due to partial epidemiological data deficiencies in the African region, many GBD predictions are based on complex statistical models rather than actual data. Such models rely on multiple assumptions and parameters, which may not fully reflect the actual disease situation. Therefore, it is recommended to conduct more population-based epidemiological studies in the African region to further improve the accuracy of GBD data. Future research in the African region should prioritize population-based epidemiological studies.

## 5 Conclusion

Our analysis highlights the burden of diabetes in Africa from 1990 to 2021, with population growth identified as the primary driver. High BMI was a risk factor for DM/T2DM DALYs, while suboptimal temperature was a risk factor for T1DM DALYs. The disparities observed among countries underscore the necessity for country-specific healthcare interventions. This will assist countries in developing more targeted prevention strategies aimed at reducing the diabetes burden and improving overall health outcomes.

## Data Availability

The datasets utilized in this research are publicly accessible through the Global Health Data Exchange (GHDx) portal: (https://vizhub.healthdata.org/gbd-results/).

## Supplementary material

SUPPLEMENTARY TABLE S1 Incidence Cases and Rates of Type 1 diabetes mellitus in African countries and estimated annual percentage changes in 1990 and 2021, along with the rankings of African countries based on age-standardized incidence rates in 1990 and 2021.

SUPPLEMENTARY TABLE S2 Incidence Cases and Rates of Type 2 diabetes mellitus in African countries and estimated annual percentage changes in 1990 and 2021, along with the rankings of African countries based on age-standardized incidence rates in 1990 and 2021.

SUPPLEMENTARY TABLE S3 Prevalence Cases and Rates of Diabetes mellitus in African countries and estimated annual percentage changes in 1990 and 2021, along with the rankings of African countries based on age-standardized prevalence rates in 1990 and 2021.

SUPPLEMENTARY TABLE S4 Prevalence Cases and Rates of Type 1 diabetes mellitus in African countries and estimated annual percentage changes in 1990 and 2021, along with the rankings of African countries based on age-standardized prevalence rates in 1990 and 2021.

SUPPLEMENTARY TABLE S5 Prevalence Cases and Rates of Type 2 diabetes mellitus in African countries and estimated annual percentage changes in 1990 and 2021, along with the rankings of African countries based on age-standardized prevalence rates in 1990 and 2021.

SUPPLEMENTARY TABLE S6 Deaths Cases and Rates of Diabetes mellitus in African countries and estimated annual percentage changes in 1990 and 2021, along with the rankings of African countries based on age-standardized deaths rates in 1990 and 2021.

SUPPLEMENTARY TABLE S7 Deaths Cases and Rates of Type 1 diabetes mellitus in African countries and estimated annual percentage changes in 1990 and 2021, along with the rankings of African countries based on age-standardized deaths rates in 1990 and 2021.

SUPPLEMENTARY TABLE S8 Deaths Cases and Rates of Type 2 diabetes mellitus in African countries and estimated annual percentage changes in 1990 and 2021, along with the rankings of African countries based on age-standardized deaths rates in 1990 and 2021.

SUPPLEMENTARY TABLE S9 Dalys Cases and Rates of Diabetes mellitus in African countries and estimated annual percentage changes in 1990 and 2021, along with the rankings of African countries based on age-standardized Dalys rates in 1990 and 2021.

SUPPLEMENTARY TABLE S10 Dalys Cases and Rates of Type 1 diabetes mellitus in African countries and estimated annual percentage changes in 1990 and 2021, along with the rankings of African countries based on age-standardized Dalys rates in 1990 and 2021.
