# Supplementary material for: African regional and national burden of diabetes mellitus and its attributable risk factors from 1990 to 2021: results from the global burden of disease study 2021
Source: Front Endocrinol (Lausanne). 2025 Aug 28;16:1643999. doi: 10.3389/fendo.2025.1643999 (PMC12422890; doi:10.3389/fendo.2025.1643999)
Supplement: SUPPLEMENTARY TABLE S1 — Incidence Cases and Rates of Type 1 diabetes mellitus in African countries and estimated annual percentage changes in 1990 and 2021, along with the rankings of African countries based on age-standardized incidence rates in 1990 and 2021. [file DataSheet1.docx]

**Table S1 | Incidence Cases and Rates of Type 1 diabetes mellitus in African countries and estimated annual percentage changes in 1990 and 2021, along with the rankings of African countries based on age-standardized incidence rates in 1990 and 2021**

| incident from Type 1 diabetes(1990-2021)in Africa | | | | | | | |
| --- | --- | --- | --- | --- | --- | --- | --- |
| location | 1990 | | | 2021 | | | EAPC 95%CI |
|  | Number(95%UI) | ASR(95%UI) | ASR Ranking | Number(95%UI) | ASR(95%UI) | ASR Ranking |  |
| Algeria | 1604.317  (1332.682-1904.270) | 5.273  (4.599-5.995) | 30 | 2842.393  (2431.970-3312.930) | 6.371  (5.481-7.440) | 20 | 0.570  (0.447-0.694) |
| Angola | 634.567  (545.942-731.526) | 5.028  (4.454-5.699) | 35 | 2080.127  (1741.971-2430.959) | 5.211  (4.554-5.951) | 38 | 0.175  (0.139-0.211) |
| Benin | 296.005  (249.875-348.942) | 5.083  (4.476-5.840) | 34 | 795.411  (678.891-936.181) | 5.078  (4.434-5.815) | 40 | 0.036  (0.016-0.057) |
| Botswana | 110.980  (92.839-130.470) | 7.817  (6.894-8.904) | 3 | 188.371  (162.636-218.880) | 8.005  (7.023-9.114) | 4 | 0.180  (0.141-0.219) |
| Burkina Faso | 590.379  (490.385-699.890) | 5.285  (4.636-6.034) | 29 | 1420.636  (1195.750-1660.455) | 5.380  (4.700-6.144) | 36 | 0.014  (-0.015-0.042) |
| Burundi | 573.960  (510.475-633.463) | 6.896  (6.211-7.617) | 13 | 1120.379  (989.866-1255.506) | 6.385  (5.714-7.089) | 19 | -0.150  (-0.187 to -0.112) |
| Cabo Verde | 18.151  (15.294-21.254) | 4.397  (3.837-5.059) | 46 | 27.676  (23.858-32.121) | 4.987  (4.313-5.712) | 43 | 0.403  (0.378-0.428) |
| Cameroon | 651.539  (555.837-761.139) | 5.348  (4.715-6.087) | 26 | 1996.187  (1716.864-2324.362) | 5.576  (4.903-6.367) | 29 | 0.117  (0.071-0.163) |
| Central African Republic | 212.270  (183.439-243.060) | 6.360  (5.694-7.101) | 19 | 403.550  (348.714-464.476) | 6.293  (5.652-7.094) | 21 | 0.008  (-0.004-0.021) |
| Chad | 327.705  (274.549-387.622) | 4.622  (4.025-5.394) | 45 | 1006.771  839.139-1214.199) | 4.683  (4.048-5.475) | 46 | 0.041  (-0.003-0.084) |
| Comoros | 42.721  (37.802-47.627) | 6.506  (5.850-7.179) | 16 | 52.487  (46.347-58.895) | 6.702  (5.959-7.480) | 14 | 0.147  (0.110-0.184) |
| Congo | 182.293  (155.313-210.663) | 6.441  (5.749-7.231) | 18 | 362.349  (317.135-419.332) | 6.252  (5.558-7.135) | 22 | 0.054  (0.009-0.099) |
| Côte d'Ivoire | 780.894  (664.601-906.763) | 5.409  (4.758-6.140) | 24 | 1737.896  (1488.227-2035.015) | 5.538  (4.863-6.347) | 30 | 0.086  (0.066-0.106) |
| Democratic Republic of the Congo | 2194.055  (1885.168-2532.434) | 4.656  (4.157-5.291) | 44 | 5157.931  (4427.407-5943.902) | 4.919  (4.326-5.608) | 44 | 0.184  (0.141-0.228) |
| Equatorial Guinea | 24.290  (20.839-27.911) | 4.712  (4.198-5.322) | 41 | 92.049  (78.323-108.126) | 5.424  (4.760-6.228) | 34 | 0.531  (0.494-0.569) |
| Eritrea | 356.209  (320.341-395.378) | 7.259  (6.551-8.021) | 8 | 576.452  (519.462-644.378) | 7.229  (6.542-8.028) | 9 | 0.036  (0.023-0.050) |
| Eswatini | 77.367  (63.804-93.956) | 8.744  (7.659-10.022) | 2 | 109.359  (92.383-128.347) | 9.472  (8.318-10.884) | 2 | 0.226  (0.179-0.273) |
| Ethiopia | 5349.959  (4785.952-5946.778) | 6.969  (6.250-7.747) | 11 | 8914.324  (7815.366-10127.715) | 6.512  (5.748-7.365) | 16 | -0.074  (-0.121- to 0.027) |
| Gabon | 71.478  (62.489-81.428) | 6.242  (5.555-6.973) | 21 | 129.997  (113.381-149.960) | 6.587  (5.834-7.438) | 15 | 0.214  (0.184-0.245) |
| Gambia | 58.047  (48.560-68.304) | 5.024  (4.387-5.740) | 36 | 145.888  (123.916-173.385) | 5.446  (4.740-6.283) | 33 | 0.245  (0.222-0.267) |
| Ghana | 826.801  (703.407-950.322) | 4.762  (4.214-5.395) | 40 | 1967.108  (1707.409-2293.752) | 5.274  (4.639-6.063) | 37 | 0.250  (0.199-0.300) |
| Guinea | 367.755  (309.484-431.898) | 5.306  (4.594-6.118) | 27 | 853.011  (716.416-1015.937) | 5.469  (4.767-6.286) | 32 | 0.063  (0.049-0.076) |
| Guinea-Bissau | 76.810  (66.008-89.095) | 6.454  (5.768-7.293) | 17 | 151.597  (129.403-175.703) | 6.443  (5.732-7.351) | 18 | 0.021  (0.008-0.034) |
| Kenya | 1682.740  (1442.512-1959.142) | 5.305  (4.677-6.061) | 28 | 3129.872  (2722.112-3686.687) | 5.695  (4.965-6.600) | 26 | 0.154  (0.100-0.209) |
| Lesotho | 127.081  (107.627-148.936) | 7.700  (6.799-8.846) | 4 | 162.689  (139.735-189.559) | 8.794  (7.714-9.960) | 3 | 0.234  (0.167-0.302) |
| Liberia | 135.444  (114.818-160.086) | 4.704  (4.096-5.423) | 42 | 306.749  (259.281-362.692) | 5.062  (4.403-5.839) | 41 | 0.158  (0.133-0.184) |
| Madagascar | 992.989  (883.061-1141.514) | 5.863  (5.240-6.566) | 23 | 1944.699  (1695.122-2224.711) | 5.603  (4.971-6.306) | 28 | -0.092  (-0.123--0.062) |
| Malawi | 1024.256  (925.921-1125.356) | 6.882  (6.261-7.559) | 14 | 1608.759  (1432.271-1797.453) | 6.775  (6.033-7.523) | 11 | 0.019  (0.000-0.039) |
| Mali | 485.343  (408.551-572.928) | 4.857  (4.212-5.623) | 39 | 1342.835  (1109.661-1591.400) | 4.762  (4.115-5.466) | 45 | -0.064  (-0.080--0.047) |
| Mauritania | 114.719  (98.409-132.422) | 4.884  (4.293-5.569) | 37 | 249.001  (210.541-294.198) | 5.108  (4.437-5.883) | 39 | 0.146  (0.112-0.180) |
| Mauritius | 123.720  (106.973-140.456) | 10.494  (9.279-11.743) | 1 | 138.523  (122.970-152.991) | 12.058  (10.953-13.310) | 1 | 0.175  (0.108-0.242) |
| Mozambique | 1343.251  (1189.276-1487.189) | 6.930  (6.234-7.572) | 12 | 3078.443  (2713.012-3467.022) | 7.260  (6.493-8.049) | 8 | 0.110  (0.070-0.149) |
| Namibia | 108.378  (91.977-126.893) | 7.341  (6.477-8.343) | 7 | 179.124  (152.553-209.007) | 7.313  (6.399-8.392) | 7 | 0.111  (0.067-0.155) |
| Niger | 408.416  (336.268-492.045) | 4.231  (3.658-4.919) | 47 | 1298.796  (1067.116-1603.103) | 4.277  (3.682-4.985) | 47 | 0.038  (0.016-0.059) |
| Nigeria | 5210.873  (4096.127-6660.817) | 5.233  (4.282-6.540) | 32 | 14598.036  (11392.005-18963.559) | 5.737  (4.657-7.146) | 24 | 0.251  (0.222-0.280) |
| Rwanda | 771.980  (695.120-847.966) | 7.387  (6.696-8.074) | 6 | 1081.775  (969.962-1212.867) | 6.979  (6.260-7.795) | 10 | -0.037  (-0.080-0.006) |
| Sao Tome and Principe | 7.119  (5.973-8.398) | 5.173  (4.554-5.893) | 33 | 13.058  (11.179-15.263) | 5.701  (5.001-6.508) | 25 | 0.290  (0.248-0.332) |
| Senegal | 477.976  (403.200-564.362) | 5.354  (4.694-6.136) | 25 | 986.870  (834.314-1162.507) | 5.630  (4.918-6.474) | 27 | 0.090  (0.072-0.107) |
| Seychelles | 5.155  (4.478-5.861) | 6.799  (6.088-7.659) | 15 | 6.790  (6.004-7.736) | 6.759  (5.968-7.725) | 12 | 0.000  (-0.034-0.034) |
| Sierra Leone | 224.356  (191.228-260.632) | 4.700  (4.127-5.365) | 43 | 498.721  (426.738-589.106) | 5.000  (4.368-5.757) | 42 | 0.110  (0.073-0.147) |
| South Africa | 2978.706  (2402.682-3779.906) | 7.582(6.214-9.397) | 5 | 4352.245  (3544.103-5411.165) | 7.761  (6.418-9.558) | 5 | 0.121  (0.038-0.203) |
| South Sudan | 504.539  (439.114-561.829) | 5.959  (5.289-6.596) | 22 | 792.743  (685.113-887.052) | 6.023  (5.307-6.724) | 23 | 0.037  (0.013-0.061) |
| Togo | 226.471  (190.941-265.193) | 5.271  (4.571-6.021) | 31 | 502.469  (434.126-587.049) | 5.481  (4.833-6.240) | 31 | 0.120  (0.088-0.152) |
| Uganda | 1702.428  (1503.179-1903.494) | 6.244  (5.594-6.994) | 20 | 3802.586  (3335.420-4240.965) | 6.448  (5.740-7.204) | 17 | 0.157  (0.143-0.172) |
| United Republic of Tanzania | 2721.054  (2329.913-3102.547) | 7.015  (6.070-7.863) | 10 | 5509.184  (4651.774-6336.356) | 7.322  (6.301-8.291) | 6 | 0.087  (0.058-0.117) |
| Zambia | 840.917  (749.761-936.323) | 7.035  (6.345-7.757) | 9 | 1659.487  (1484.839-1855.665) | 6.707  (6.029-7.438) | 13 | -0.044  (-0.077 to -0.010) |
| Zimbabwe | 597.535  (496.590-709.859) | 4.870  (4.214-5.613) | 38 | 932.690  (784.383-1086.192) | 5.381  (4.653-6.106) | 35 | 0.131  (0.039-0.223) |

**Table S2 | Incidence Cases and Rates of Type 2 diabetes mellitus in African countries and estimated annual percentage changes in 1990 and 2021, along with the rankings of African countries based on age-standardized incidence rates in 1990 and 2021**

| incident from Type 2 diabetes(1990-2021)in Africa | | | | | | | |
| --- | --- | --- | --- | --- | --- | --- | --- |
| location | 1990 | | | 2021 | | | EAPC 95%CI |
|  | Number(95%UI) | ASR(95%UI) | ASR Ranking | Number(95%UI) | ASR(95%UI) | ASR Ranking |  |
| Algeria | 32728.665  (29735.880-35985.613) | 207.320  (186.614-230.871) | 6 | 209798.708  (192595.327-226848.866) | 478.548  (443.006-516.404) | 3 | 2.767  (2.739-2.795) |
| Angola | 10714.242  (9815.296-11798.656) | 195.359  (179.893-212.678) | 9 | 60603.337  (55421.507-66669.381) | 301.367  (275.924-327.496) | 14 | 1.469  (1.433-1.505) |
| Benin | 4165.196  (3796.525-4558.345) | 165.282  (149.627-180.715) | 18 | 25730.555  (23498.396-28311.426) | 307.017  (280.872-337.435) | 13 | 1.915  (1.823-2.007) |
| Botswana | 1164.325  (1070.930-1262.991) | 175.196  (161.043-189.484) | 13 | 5649.319  (5251.006-6062.230) | 295.641  (277.019-314.275) | 15 | 1.785  (1.744-1.827) |
| Burkina Faso | 6648.777  (6173.449-7186.784) | 132.841  (123.858-142.248) | 35 | 30211.541  (27648.469-32619.599) | 227.174  (208.647-243.557) | 32 | 1.755  (1.659-1.850) |
| Burundi | 3716.672  (3469.589-3996.098) | 132.779  (124.113-142.769) | 36 | 12090.979  (11144.034-13030.277) | 169.526  (156.644-182.333) | 41 | 0.717  (0.683-0.750) |
| Cabo Verde | 369.700  (336.710-404.763) | 152.434  (138.957-167.140) | 23 | 1770.048  (1616.484-1936.052) | 331.173  (302.716-361.048) | 11 | 2.743  (2.627-2.860) |
| Cameroon | 8316.365  (7600.764-9043.973) | 149.004  (138.189-160.781) | 24 | 50577.385  (46784.347-54823.291) | 258.514  (240.909-277.716) | 23 | 1.878  (1.788-1.968) |
| Central African Republic | 3363.470  (3082.602-3684.769) | 212.721  (195.171-232.712) | 4 | 13094.952  (11931.219-14247.973) | 335.106  (307.162-362.846) | 9 | 1.509  (1.467-1.551) |
| Chad | 4626.076  (4246.749-5041.210) | 135.992  (124.684-148.649) | 34 | 22472.515  (20484.750-24504.409) | 241.943  (220.449-263.210) | 28 | 1.841  (1.691-1.991) |
| Comoros | 375.446  (347.733-409.076) | 159.971  (148.326-174.701) | 19 | 1422.808  (1317.900-1541.059) | 236.080  (219.080-255.873) | 31 | 1.283  (1.254-1.312) |
| Congo | 2481.591  (2282.505-2680.849) | 184.592  (172.178-196.893) | 11 | 12046.727  (11036.999-13158.778) | 294.770  (273.583-318.228) | 16 | 1.577  (1.535-1.618) |
| Côte d'Ivoire | 9491.777  (8610.349-10381.517) | 158.970  (146.089-172.312) | 20 | 48817.841  (44501.431-53313.826) | 274.186  (251.520-301.244) | 19 | 1.806  (1.755-1.856) |
| Democratic Republic of the Congo | 31632.778  (29126.251-34406.263) | 156.831  (144.712-169.336) | 21 | 140622.278  (128003.279-153553.258) | 239.205  (218.788-258.267) | 29 | 1.389  (1.340-1.438) |
| Equatorial Guinea | 428.511  (395.272-464.529) | 176.167  (163.595-190.560) | 12 | 3047.557  (2782.124-3338.263) | 331.492  (308.550-356.219) | 10 | 2.280  (2.192-2.368) |
| Eritrea | 2212.958  (2031.895-2424.812) | 145.783  (134.361-158.044) | 25 | 9513.459  (8728.389-10307.715) | 220.886  (204.112-238.003) | 34 | 1.360  (1.339-1.382) |
| Eswatini | 792.076  (735.359-859.549) | 225.689  (210.773-243.003) | 2 | 2998.033  (2801.788-3209.450) | 408.343  (383.461-433.489) | 4 | 2.081  (1.970-2.191) |
| Ethiopia | 42886.459  (39190.279-46895.697) | 170.928  (156.413-186.988) | 17 | 118170.169  (107636.788-129257.290) | 187.791  (171.513-205.641) | 38 | 0.151  (0.073-0.229) |
| Gabon | 1361.325  (1261.272-1476.561) | 208.641  (194.247-224.624) | 5 | 5110.287  (4742.559-5480.558) | 358.356  (335.241-382.803) | 7 | 1.840  (1.769-1.910) |
| Gambia | 678.975  (616.493-740.137) | 138.290  (125.820-151.444) | 32 | 3832.474  (3514.947-4187.861) | 260.668  (238.770-286.024) | 22 | 2.144  (2.100-2.187) |
| Ghana | 12095.865  (11015.066-13225.795) | 143.359  (131.767-155.871) | 26 | 64873.603  (58902.547-70351.435) | 265.780  (243.780-289.574) | 20 | 2.022  (1.844-2.200) |
| Guinea | 4956.153  (4565.805-5391.198) | 131.555  (121.572-143.323) | 37 | 18059.995  (16698.267-19586.328) | 224.833  (207.480-243.121) | 33 | 1.585  (1.501-1.670) |
| Guinea-Bissau | 934.389  (846.684-1016.228) | 173.852  (159.214-188.993) | 14 | 3652.776  (3340.887-3985.051) | 292.787  (269.876-317.582) | 17 | 1.715  (1.682-1.749) |
| Kenya | 10151.054  (9282.882-11086.142) | 102.227  (93.880-112.088) | 47 | 39803.019  (36531.611-43542.855) | 134.555  (123.503-147.532) | 47 | 0.857  (0.794-0.920) |
| Lesotho | 1307.678  (1216.365-1406.736) | 141.788  (132.710-152.334) | 28 | 4187.081  (3930.993-4448.268) | 315.987  (297.681-334.375) | 12 | 2.889  (2.801-2.978) |
| Liberia | 2188.642  (2009.190-2393.757) | 153.780  (140.850-168.250) | 22 | 10020.649  (9071.376-10968.515) | 278.446  (253.921-303.424) | 18 | 2.022  (1.984-2.061) |
| Madagascar | 6615.207  (6105.314-7165.186) | 110.188  (102.277-119.494) | 45 | 26038.089  (23821.700-28161.719) | 155.517  (143.132-167.623) | 44 | 1.097  (1.079-1.115) |
| Malawi | 5001.550  (4662.290-5382.724) | 114.547  (107.880-122.056) | 44 | 13990.560  (13074.064-15048.220) | 142.258  (133.280-151.595) | 46 | 0.631  (0.601-0.662) |
| Mali | 11544.462  (10394.413-12683.555) | 224.891  (203.268-246.320) | 3 | 57448.025  (52473.563-63655.364) | 405.138  (370.916-444.052) | 5 | 1.962  (1.890-2.035) |
| Mauritania | 1543.471  (1431.499-1665.764) | 129.314  (120.250-139.449) | 38 | 5198.521  (4902.244-5461.805) | 183.989  (174.101-192.643) | 39 | 1.092  (0.955-1.229) |
| Mauritius | 2722.628  (2550.198-2920.359) | 307.256  (286.539-330.593) | 1 | 11383.125  (10642.604-12184.581) | 629.446  (591.110-672.285) | 1 | 2.378  (2.228-2.528) |
| Mozambique | 7880.007  (7342.957-8439.799) | 119.402  (112.702-127.177) | 41 | 31561.746  (28923.381-34280.960) | 193.911  (179.896-207.059) | 36 | 1.673  (1.623-1.724) |
| Namibia | 1310.843  (1212.141-1426.988) | 173.814  (161.452-187.433) | 15 | 4278.860  (3906.581-4666.476) | 242.476  (222.608-263.591) | 27 | 1.075  (1.055-1.095) |
| Niger | 5506.793  (4997.750-6008.730) | 141.131  (128.523-153.138) | 29 | 31325.626  (28433.369-34496.389) | 238.773  (217.398-261.043) | 30 | 1.725  (1.708-1.741) |
| Nigeria | 72729.313  (66091.555-79798.230) | 136.369  (124.110-150.578) | 33 | 267508.618  (243565.170-291800.469) | 203.054  (185.698-222.000) | 35 | 1.257  (1.215-1.298) |
| Rwanda | 4233.161  (3921.417-4546.095) | 128.532  (120.647-136.524) | 39 | 11919.047  (10965.041-12885.789) | 146.826  (136.256-157.129) | 45 | 0.277  (0.189-0.365) |
| Sao Tome and Principe | 105.432  (95.683-115.801) | 140.480  (126.754-154.674) | 30 | 435.346  (394.813-481.277) | 262.753  (237.832-289.528) | 21 | 2.049  (2.035-2.063) |
| Senegal | 8261.151  (7512.904-9044.385) | 198.593  (179.191-217.325) | 8 | 38283.888  (35644.605-41046.036) | 361.543  (336.005-391.428) | 6 | 2.088  (1.960-2.216) |
| Seychelles | 121.313  (111.153-133.135) | 206.017  (186.971-227.053) | 7 | 751.883  (691.788-816.147) | 586.534  (543.501-633.594) | 2 | 3.449  (3.273-3.625) |
| Sierra Leone | 3497.093  (3193.845-3830.190) | 139.104  (126.246-152.096) | 31 | 14293.771  (12990.735-15591.687) | 248.197  (227.337-271.141) | 25 | 1.940  (1.901-1.980) |
| South Africa | 47439.624  (43331.366-52085.635) | 193.584  (176.330-213.075) | 10 | 181422.184  (166528.549-198201.625) | 338.773  (311.449-368.898) | 8 | 1.954  (1.898-2.010) |
| South Sudan | 3580.323  (3316.857-3892.447) | 118.836  (110.503-128.836) | 42 | 8928.757  (8191.123-9698.174) | 167.625  (155.682-180.142) | 42 | 1.096  (1.079-1.113) |
| Togo | 1983.083  (1803.491-2157.629) | 115.543  (106.846-125.470) | 43 | 10525.079  (9742.233-11324.209) | 188.360  (173.864-202.540) | 37 | 1.596  (1.580-1.613) |
| Uganda | 9214.251  (8543.062-9920.436) | 121.455  (113.133-130.783) | 40 | 36796.008  (33858.652-39633.021) | 175.091  (162.565-187.697) | 40 | 1.164  (1.127-1.202) |
| United Republic of Tanzania | 12990.329  (12071.018-13897.756) | 105.730  (99.548-112.148) | 46 | 53513.679  (49814.757-57367.568) | 162.329  (151.746-172.596) | 43 | 1.416  (1.374-1.459) |
| Zambia | 6221.290  (5719.593-6726.990) | 171.872  (159.302-186.483) | 16 | 27696.418  (25463.001-30172.873) | 244.919  (226.354-265.296) | 26 | 1.122  (1.101-1.142) |
| Zimbabwe | 7107.907  6507.203-7703.555) | 142.714  (132.534-155.404) | 27 | 23775.106  (21994.818-25727.374) | 249.004  (230.846-268.162) | 24 | 1.981  (1.931-2.032) |

**Table S3 | Prevalence Cases and Rates of Diabetes mellitus in African countries and estimated annual percentage changes in 1990 and 2021, along with the rankings of African countries based on age-standardized prevalence rates in 1990 and 2021**

| Prevalence from diabetes mellitus(1990-2021) in the African region | | | | | | | |
| --- | --- | --- | --- | --- | --- | --- | --- |
| location | 1990 | | | 2021 | | | EAPC 95%CI |
|  | Number(95%UI) | ASR(95%UI) | ASR  Ranking | Number(95%UI) | ASR(95%UI) | ASR Ranking |  |
| Algeria | 563378.632  (513630.032-616216.262) | 3981.684  (3607.064-4371.577) | 3 | 3984403.312  (3692175.188-4266453.085) | 10017.152  (9269.636-10746.274) | 3 | 3.021  (2.998-3.044) |
| Angola | 169979.766  (155437.673-186909.124) | 3280.591  (3004.532-3596.973) | 7 | 1050811.870  (956260.230-1156436.898) | 6181.636  (5660.110-6718.484) | 12 | 2.167  (2.103-2.232) |
| Benin | 72400.128  (66082.392-78935.914) | 2953.303  (2682.909-3217.271) | 11 | 476046.057  (437987.075-517026.257) | 6560.089  (6018.815-7124.429) | 9 | 2.477  (2.346-2.608) |
| Botswana | 15938.908  (14554.327-17545.021) | 2388.049  (2180.394-2618.383) | 27 | 84885.981  (78196.999-92012.607) | 4855.881  (4484.182-5231.668) | 25 | 2.396  (2.369-2.422) |
| Burkina Faso | 98877.153  (91031.391-107179.175) | 1975.343  (1822.486-2142.468) | 36 | 533083.857  (488956.452-579086.095) | 4400.238  (4039.747-4725.281) | 31 | 2.638  (2.534-2.742) |
| Burundi | 54293.206  (50385.771-58952.491) | 1826.218  (1695.358-1981.993) | 39 | 199594.800  (183086.397-215681.810) | 2924.323  (2688.803-3154.477) | 40 | 1.464  (1.439-1.489) |
| Cabo Verde | 6954.535  (6370.202-7538.151) | 2911.154  (2669.502-3165.251) | 12 | 35035.727  (32230.551-37961.800) | 6967.365  (6431.970-7547.687) | 7 | 3.051  (2.904-3.199) |
| Cameroon | 132475.501  (120020.373-143468.759) | 2408.302  (2179.623-2609.302) | 26 | 883052.938  (809703.773-962366.238) | 5020.835  (4651.645-5425.586) | 22 | 2.458  (2.360-2.557) |
| Central African Republic | 54097.606  (49143.780-59317.568) | 3674.309  (3358.726-4008.012) | 4 | 229275.765  (209217.312-249365.665) | 7074.487  (6481.850-7656.482) | 6 | 2.198  (2.132-2.264) |
| Chad | 78657.341  (71755.449-85474.468) | 2378.208  (2177.956-2579.489) | 29 | 394373.307  (361360.443-429328.155) | 4877.644  (4458.054-5293.802) | 24 | 2.287  (2.134-2.440) |
| Comoros | 5663.452  (5232.235-6147.269) | 2360.224  (2180.032-2566.329) | 30 | 24977.639  (23148.863-27029.624) | 4405.056  (4084.641-4761.247) | 29 | 2.076  (2.044-2.107) |
| Congo | 36233.781  (32825.387-39387.029) | 2783.144  (2535.861-3025.261) | 15 | 204389.022  (186645.047-222614.394) | 5692.930  (5251.841-6176.299) | 17 | 2.362  (2.321-2.403) |
| Côte d'Ivoire | 158329.995  (143720.853-173234.678) | 2743.049  (2490.180-2971.398) | 17 | 871359.457  (796794.067-941681.535) | 5514.637  (5067.990-5957.579) | 18 | 2.299  (2.241-2.357) |
| Democratic Republic of the Congo | 491813.568  (450849.798-539209.279) | 2538.284  (2324.301-2773.626) | 21 | 2418160.736  (2208525.281-2634393.872) | 4749.033  (4351.915-5143.966) | 27 | 1.996  (1.954-2.039) |
| Equatorial Guinea | 6680.250  (6099.006-7264.520) | 2884.053  (2645.263-3144.477) | 14 | 49763.015  (45442.360-54306.780) | 6373.133  (5827.329-6881.770) | 11 | 2.747  (2.684-2.811) |
| Eritrea | 34826.150  (31817.306-37927.202) | 2147.611  (1970.518-2347.234) | 33 | 161355.152  (147950.801-176759.653) | 4064.455  (3724.749-4385.303) | 34 | 2.103  (2.079-2.128) |
| Eswatini | 10298.649  (9466.587-11230.539) | 2888.631  (2651.520-3175.402) | 13 | 42528.066  (39385.725-46043.083) | 6396.383  (5917.894-6924.267) | 10 | 2.768  (2.657-2.879) |
| Ethiopia | 645481.279  (586011.577-705373.024) | 2499.733  (2258.955-2740.110) | 22 | 2005665.453  (1810001.607-2198428.105) | 3357.888  (3054.953-3688.035) | 38 | 0.801  (0.748-0.854) |
| Gabon | 20712.265  (18984.528-22564.707) | 3268.835  (3010.037-3562.501) | 8 | 86729.320  (80044.243-94478.012) | 6787.112  (6298.393-7329.148) | 8 | 2.414  (2.337-2.492) |
| Gambia | 11563.177  (10477.679-12644.274) | 2424.046  (2216.380-2633.533) | 25 | 68867.878  (63235.057-75047.789) | 5217.712  (4821.390-5645.699) | 20 | 2.555  (2.505-2.605) |
| Ghana | 206852.728  (188337.301-226136.526) | 2557.494  (2328.728-2781.577) | 19 | 1174351.182  (1069046.398-1268670.095) | 5329.954  (4879.695-5757.845) | 19 | 2.414  (2.285-2.544) |
| Guinea | 82540.882  (75680.672-89573.025) | 2221.720  (2032.182-2416.370) | 31 | 323491.741  (298813.593-349827.915) | 4402.961  (4068.740-4758.924) | 30 | 2.079  (2.007-2.151) |
| Guinea-Bissau | 15661.052  (14263.643-17130.860) | 3035.654  (2758.011-3317.771) | 10 | 64230.601  (59087.062-69910.765) | 5949.506  (5504.348-6455.568) | 13 | 2.186  (2.133-2.240) |
| Kenya | 152096.092  (138632.563-165216.885) | 1398.882  (1278.829-1532.822) | 46 | 596808.764  (542712.939-650178.396) | 2044.356  (1872.372-2231.736) | 47 | 1.143  (1.084-1.202) |
| Lesotho | 17273.057  (15730.219-18836.400) | 1848.796  (1683.814-2024.513) | 38 | 60225.234  (55564.512-65205.423) | 4884.666  (4518.535-5280.633) | 23 | 3.398  (3.312-3.485) |
| Liberia | 37207.608  (34021.324-40470.520) | 2693.404  (2473.396-2931.074) | 18 | 181656.440  (165251.316-200450.673) | 5788.187  (5296.516-6282.547) | 16 | 2.589  (2.523-2.654) |
| Madagascar | 103096.313  (94943.700-111643.263) | 1610.614  (1481.670-1743.820) | 41 | 429036.434  (393072.329-467930.447) | 2748.514  (2526.164-2985.533) | 43 | 1.709  (1.691-1.726) |
| Malawi | 70032.611  (64822.485-75841.136) | 1418.297  (1315.116-1526.859) | 45 | 212859.031  (198366.855-228304.427) | 2110.477  (1954.407-2265.954) | 46 | 1.185  (1.132-1.238) |
| Mali | 205259.591  (187792.616-224711.545) | 4324.801  (3964.334-4717.727) | 2 | 1048006.322  (955978.882-1141820.393) | 8756.389  (8016.779-9542.349) | 4 | 2.407  (2.266-2.549) |
| Mauritania | 24435.374  (22509.370-26555.814) | 2074.971  (1903.275-2252.005) | 35 | 90230.205  (84605.267-95571.405) | 3432.692  (3230.991-3620.456) | 37 | 1.504  (1.358-1.650) |
| Mauritius | 42958.480  (40253.702-46205.942) | 5362.414  (5028.878-5766.079) | 1 | 201711.475  (187180.933-216219.250) | 11198.412  (10450.807-11952.101) | 1 | 2.443  (2.316-2.571) |
| Mozambique | 112831.742  (104008.884-121895.699) | 1554.357  (1434.348-1670.485) | 44 | 507374.800  (464368.108-554016.408) | 3280.146  (2995.542-3568.910) | 39 | 2.544  (2.506-2.582) |
| Namibia | 17906.529  (16324.345-19702.490) | 2384.395  (2181.738-2623.493) | 28 | 66849.965  (60937.193-73139.949) | 4112.688  (3743.112-4509.605) | 33 | 1.795  (1.766-1.823) |
| Niger | 92667.050  (84461.305-100889.235) | 2478.351  (2269.025-2690.744) | 23 | 547427.507  (499050.105-597510.007) | 4842.017  (4428.238-5296.631) | 26 | 2.186  (2.160-2.212) |
| Nigeria | 1154539.126  (1046974.701-1259282.787) | 2219.389  (2010.161-2439.102) | 32 | 4415640.945  (4004326.784-4821587.519) | 3695.541  (3382.956-4032.829) | 35 | 1.617  (1.581-1.653) |
| Rwanda | 59560.821  (55083.113-64474.425) | 1614.269  (1492.954-1746.431) | 40 | 185581.227  (171587.121-200808.166) | 2318.135  (2137.745-2491.664) | 45 | 0.981  (0.889-1.073) |
| Sao Tome and Principe | 1971.099  (1800.898-2147.382) | 2744.041  (2512.119-2988.212) | 16 | 8605.629  (7943.013-9401.621) | 5843.305  (5388.095-6334.538) | 15 | 2.484  (2.469-2.500) |
| Senegal | 142797.823  (130243.986-155641.803) | 3618.211  (3305.005-3927.964) | 5 | 676255.537  (631202.935-721960.988) | 7138.583  (6681.317-7626.763) | 5 | 2.383  (2.255-2.512) |
| Seychelles | 2054.399  (1881.657-2249.648) | 3528.484  (3228.909-3867.492) | 6 | 13181.481  (12166.936-14257.119) | 10992.129  (10199.143-11825.159) | 2 | 3.707  (3.522-3.892) |
| Sierra Leone | 60010.846  (54564.714-65583.814) | 2446.548  (2221.139-2683.190) | 24 | 261588.530  (239208.223-285980.525) | 5087.884  (4659.147-5497.924) | 21 | 2.438  (2.387-2.489) |
| South Africa | 735879.040  (663364.409-808232.136) | 3066.897  (2771.740-3400.398) | 9 | 2945186.154  (2686543.389-3243894.412) | 5866.548  (5350.976-6451.323) | 14 | 2.212  (2.172-2.253) |
| South Sudan | 51006.680  (46628.465-55544.076) | 1610.209  (1473.156-1757.437) | 42 | 145174.158  (133597.279-157084.152) | 2859.558  (2636.959-3082.370) | 41 | 1.856  (1.839-1.872) |
| Togo | 32826.387  (29828.785-35726.235) | 1915.880  (1750.041-2082.547) | 37 | 182191.393  (169167.046-196649.225) | 3592.501  (3350.650-3852.419) | 36 | 2.034  (1.996-2.071) |
| Uganda | 131842.542  (121274.415-142762.411) | 1589.032  (1459.229-1729.968) | 43 | 581230.718  (535241.736-630152.105) | 2822.270  (2585.308-3056.423) | 42 | 1.814  (1.766-1.861) |
| United Republic of Tanzania | 187814.362  (173373.553-202375.801) | 1368.985  (1272.887-1467.261) | 47 | 864057.532  (805665.613-926503.624) | 2634.932  (2454.331-2815.217) | 44 | 2.150  (2.104-2.195) |
| Zambia | 96607.598  (88957.223-104080.590) | 2554.496  (2366.655-2758.165) | 20 | 478452.317  (439036.038-522496.010) | 4617.710  (4269.802-5001.267) | 28 | 1.926  (1.903-1.950) |
| Zimbabwe | 104120.096  (94820.583-112930.756) | 2092.047  (1911.279-2267.092) | 34 | 372979.319  (343640.382-406058.684) | 4328.416  (4004.574-4712.405) | 32 | 2.510  (2.462-2.557) |

**Table S4 | Prevalence Cases and Rates of Type 1 diabetes mellitus in African countries and estimated annual percentage changes in 1990 and 2021, along with the rankings of African countries based on age-standardized prevalence rates in 1990 and 2021**

| Prevalence from Type 1 diabetes(1990-2021)in Africa | | | | | | | |
| --- | --- | --- | --- | --- | --- | --- | --- |
| location | 1990 | | | 2021 | | | EAPC 95%CI |
|  | Number(95%UI) | ASR(95%UI) | ASR Ranking | Number(95%UI) | ASR(95%UI) | ASR Ranking |  |
| Algeria | 36911.696  (29976.910-44767.779) | 175.562  (146.524-206.264) | 25 | 92390.634  (76247.397-111679.455) | 211.641  (174.276-255.559) | 16 | 0.559  (0.423-0.695) |
| Angola | 13021.598  (10914.423-15524.466) | 151.189  (128.821-177.623) | 35 | 42523.840  (35195.014-50895.717) | 156.351  (131.616-184.841) | 37 | 0.164  (0.125-0.203) |
| Benin | 5640.996  (4636.938-6788.892) | 150.723  (127.138-178.309) | 36 | 16579.953  (13694.887-20287.919) | 151.420  (127.745-181.614) | 41 | 0.055  (0.033-0.077) |
| Botswana | 2340.131  (1870.400-2808.471) | 221.311  (182.692-263.406) | 12 | 5256.044  (4338.686-6335.422) | 226.877  (188.936-272.029) | 10 | 0.191  (0.143-0.238) |
| Burkina Faso | 11412.446  (9393.708-13833.573) | 153.656  (129.284-183.133) | 31 | 28986.271  (23900.424-34866.778) | 157.633  (131.775-189.030) | 36 | 0.048  (0.019-0.078) |
| Burundi | 11752.231  (10312.888-13142.998) | 230.423  (203.842-258.122) | 10 | 26331.479  (22894.545-29929.413) | 215.254  (186.756-243.912) | 15 | -0.097  (-0.159 to -0.035) |
| Cabo Verde | 393.730  (325.998-468.727) | 136.777  (116.168-162.231) | 45 | 875.715  (731.350-1046.325) | 153.968  (129.523-182.810) | 39 | 0.399  (0.368-0.429) |
| Cameroon | 13077.616  (10858.099-15606.468) | 156.333  (132.090-183.543) | 28 | 43526.754  (36313.843-52272.643) | 162.990  (139.420-194.320) | 31 | 0.118  (0.064-0.173) |
| Central African Republic | 4319.970  (3601.496-5082.536) | 183.057  (154.054-214.448) | 22 | 8722.285  (7199.189-10461.378) | 177.754  (150.495-208.585) | 25 | -0.046  (-0.071 to -0.021) |
| Chad | 6393.701  (5241.873-7755.396) | 135.933  (113.753-163.371) | 46 | 18653.198  (15266.305-22979.649) | 137.924  (115.827-165.547) | 46 | 0.048  (-0.003-0.099) |
| Comoros | 945.960  (828.670-1070.511) | 223.278  (196.065-252.018) | 11 | 1663.041  (1443.510-1886.950) | 227.285  (197.464-257.204) | 9 | 0.115  (0.060-0.170) |
| Congo | 3779.647  (3099.987-4551.976) | 184.612  (154.884-218.989) | 21 | 9071.638  (7483.800-10714.089) | 181.395  (153.133-213.737) | 24 | 0.091  (0.043-0.139) |
| Côte d'Ivoire | 15521.404  (12769.392-18493.172) | 158.989  (134.876-188.602) | 26 | 38706.313  (31920.126-46135.850) | 162.782  (137.699-193.384) | 32 | 0.109  (0.081-0.137) |
| Democratic Republic of the Congo | 44376.339  (37408.021-52179.243) | 140.257  (119.581-163.946) | 41 | 115585.609  (95919.823-136904.351) | 148.166  (125.536-173.593) | 44 | 0.190  (0.147-0.233) |
| Equatorial Guinea | 483.354  (404.186-566.437) | 137.896  (117.643-160.824) | 44 | 2167.842  (1788.771-2599.051) | 163.987  (138.812-193.856) | 30 | 0.676  (0.626-0.726) |
| Eritrea | 7633.492  (6763.896-8536.851) | 241.706  (214.512-273.354) | 5 | 15310.619  (13582.824-17331.842) | 240.219  (212.730-272.492) | 5 | 0.041  (0.013-0.069) |
| Eswatini | 1526.584  (1234.081-1887.303) | 245.202  (203.046-294.805) | 2 | 2654.762  (2136.407-3211.262) | 259.451  (214.090-308.333) | 2 | 0.156  (0.097-0.216) |
| Ethiopia | 111816.861  (99214.590-124656.508) | 238.384  (212.570-267.969) | 6 | 230445.950  (201045.435-260268.611) | 225.706  (197.767-256.403) | 12 | -0.032  (-0.092-0.029) |
| Gabon | 1592.698  (1347.625-1859.360) | 185.026  (157.930-216.138) | 20 | 3313.832  (2789.987-3965.956) | 195.627  (165.640-231.022) | 19 | 0.223  (0.193-0.253) |
| Gambia | 1157.645  (941.796-1400.186) | 149.384  (124.780-177.210) | 37 | 3207.306  (2618.079-3884.537) | 160.150  (134.908-192.193) | 34 | 0.215  (0.186-0.243) |
| Ghana | 17310.754  (14260.201-20521.792) | 141.195  (120.296-164.990) | 40 | 47384.677  (40098.774-56555.342) | 155.876  (133.062-184.279) | 38 | 0.239  (0.189-0.289) |
| Guinea | 7659.981  (6335.912-9048.456) | 156.001  (130.054-184.936) | 29 | 17813.370  (14540.179-21631.312) | 161.065  (134.503-193.172) | 33 | 0.072  (0.060-0.085) |
| Guinea-Bissau | 1479.925  (1229.503-1770.348) | 182.551  (155.149-213.427) | 23 | 3140.243  (2569.527-3776.854) | 181.573  (153.108-214.735) | 23 | 0.015  (-0.001-0.031) |
| Kenya | 35245.835  (30088.510-41684.143) | 176.405  (152.325-206.389) | 24 | 87060.997  (73858.731-103995.941) | 185.864  (158.721-219.380) | 22 | 0.103  (0.061-0.146) |
| Lesotho | 2731.659  (2250.316-3258.330) | 215.641  (180.419-256.517) | 14 | 4067.067  (3326.818-4879.395) | 238.062  (198.866-284.293) | 6 | 0.145  (0.080-0.210) |
| Liberia | 2766.367  (2270.457-3346.172) | 138.819  (116.624-165.663) | 43 | 7074.841  (5836.204-8459.442) | 150.227  (125.363-178.653) | 42 | 0.208  (0.185-0.231) |
| Madagascar | 21573.016  (18796.047-25101.234) | 198.899  (175.153-229.130) | 18 | 49611.225  (42672.932-58325.341) | 186.642  (160.963-215.437) | 21 | -0.135  (-0.190 to -0.081) |
| Malawi | 21007.503  (18684.908-23340.820) | 233.568  (207.727-261.054) | 9 | 41607.039  (36729.585-46923.045) | 227.364  (200.647-256.183) | 8 | -0.005  (-0.045-0.034) |
| Mali | 9781.745  (8043.621-11844.723) | 142.502  (119.759-171.396) | 39 | 26457.883  (21375.680-32372.946) | 141.619  (118.029-168.023) | 45 | -0.020  (-0.037 to -0.003) |
| Mauritania | 2406.738  (2018.330-2841.276) | 144.383  (123.122-170.878) | 38 | 5639.841  (4646.242-6820.055) | 153.796  (129.693-183.913) | 40 | 0.200  (0.164-0.235) |
| Mauritius | 3498.183  (2715.689-4219.860) | 316.895  (253.555-375.536) | 1 | 4908.421  (4221.938-5640.424) | 329.140  (279.370-381.621) | 1 | -0.102  (-0.174 to -0.031) |
| Mozambique | 29275.891  (25888.788-32712.660) | 235.567  (207.242-264.283) | 8 | 70295.323  (61579.760-79810.674) | 242.204  (212.851-275.468) | 4 | 0.071  (-0.004-0.146) |
| Namibia | 2353.853  (1923.004-2846.143) | 206.548  (173.275-248.069) | 17 | 4630.649  (3741.326-5594.750) | 209.427  (173.656-250.833) | 18 | 0.147  (0.097-0.198) |
| Niger | 7689.738  (6173.996-9401.146) | 125.062  (104.045-150.109) | 47 | 24167.954  (19312.134-30080.955) | 128.399  (107.290-154.304) | 47 | 0.095  (0.070-0.120) |
| Nigeria | 111571.953  (86619.107-145070.381) | 152.327  (119.967-194.658) | 34 | 307598.618  (236158.454-404488.230) | 166.287  (131.510-212.832) | 28 | 0.248  (0.215-0.281) |
| Rwanda | 16121.479  (14313.004-18000.512) | 243.490  (216.325-271.509) | 4 | 29856.405  (26376.107-33610.509) | 234.846  (208.376-264.725) | 7 | 0.042  (-0.013-0.097) |
| Sao Tome and Principe | 148.431  (122.633-177.583) | 153.565  (129.779-183.348) | 32 | 329.265  (270.131-396.253) | 168.973  (142.093-200.714) | 26 | 0.293  (0.250-0.336) |
| Senegal | 9465.904  (7810.582-11593.915) | 158.184  (133.307-189.605) | 27 | 22721.319  (18694.925-27620.898) | 166.885  (139.649-199.919) | 27 | 0.096  (0.078-0.115) |
| Seychelles | 125.191  (104.270-147.906) | 186.927  (157.292-219.731) | 19 | 220.386  (183.433-262.218) | 187.691  (156.152-221.820) | 20 | 0.029  (0.002-0.055) |
| Sierra Leone | 4780.707  (3966.279-5710.028) | 139.467  (118.002-164.483) | 42 | 11224.133  (9320.159-13671.523) | 148.284  (125.755-177.559) | 43 | 0.122  (0.086-0.158) |
| South Africa | 71860.488  (56183.897-92521.513) | 220.467  (175.315-278.701) | 13 | 128426.655  (101219.215-163774.429) | 223.058  (177.200-282.528) | 13 | 0.098  (0.011-0.185) |
| South Sudan | 11660.835  (10046.462-13090.069) | 213.464  (184.837-240.333) | 15 | 18984.466  (16297.984-21397.016) | 209.894  (180.599-237.643) | 17 | -0.030  (-0.080-0.020) |
| Togo | 4393.436  (3621.649-5362.789) | 154.550  (130.084-183.037) | 30 | 11712.128  (9728.456-13971.460) | 160.078  (135.911-190.129) | 35 | 0.118  (0.082-0.154) |
| Uganda | 32931.672  (28761.894-37168.040) | 213.046  (187.272-241.611) | 16 | 86377.957  (75105.341-97751.897) | 217.547  (190.067-247.474) | 14 | 0.126  (0.106-0.145) |
| United Republic of Tanzania | 58255.934  (48639.885-66889.979) | 244.633  (206.490-281.063) | 3 | 139490.560  (115555.470-163948.331) | 252.900  (210.507-296.344) | 3 | 0.054  (0.007-0.101) |
| Zambia | 17333.439  (15275.472-19411.319) | 237.622  (209.706-265.454) | 7 | 41437.648  (36410.299-46716.563) | 225.908  (199.288-257.034) | 11 | -0.043  (-0.080 to -0.005) |
| Zimbabwe | 12653.429  (10270.013-15334.502) | 152.490  (126.816-181.388) | 33 | 22287.479  (18242.208-26860.235) | 164.127  (135.046-194.032) | 29 | 0.026  (-0.066-0.117) |

**Table S5 | Prevalence Cases and Rates of Type 2 diabetes mellitus in African countries and estimated annual percentage changes in 1990 and 2021, along with the rankings of African countries based on age-standardized prevalence rates in 1990 and 2021**

| Prevalence from Type 2 diabetes(1990-2021)in Africa | | | | | | | |
| --- | --- | --- | --- | --- | --- | --- | --- |
| location | 1990 | | | 2021 | | | EAPC 95%CI |
|  | Number(95%UI) | ASR(95%UI) | ASR Ranking | Number(95%UI) | ASR(95%UI) | ASR Ranking |  |
| Algeria | 526466.936  (474101.171-582049.417) | 3806.122  (3425.265-4203.129) | 3 | 3892012.679  (3591807.704-4171456.817) | 9805.511  (9057.656-10519.540) | 3 | 3.098  (3.079-3.117) |
| Angola | 156958.168  (142953.208-174230.276) | 3129.402  (2863.878-3446.897) | 7 | 1008288.030  (911815.376-1115010.102) | 6025.285  (5499.410-6570.407) | 12 | 2.239  (2.172-2.307) |
| Benin | 66759.131  (60385.747-73248.898) | 2802.580  (2530.480-3070.114) | 11 | 459466.104  (421648.274-500582.249) | 6408.670  (5865.695-6974.388) | 9 | 2.566  (2.431-2.701) |
| Botswana | 13598.777  (12122.111-15147.096) | 2166.738  (1961.719-2396.607) | 29 | 79629.937  (72956.861-86816.852) | 4629.004  (4250.285-4988.322) | 26 | 2.554  (2.527-2.581) |
| Burkina Faso | 87464.707  (79297.983-95779.525) | 1821.688  (1661.835-1988.280) | 36 | 504097.586  (458181.772-549809.976) | 4242.605  (3887.294-4576.488) | 29 | 2.791  (2.686-2.896) |
| Burundi | 42540.975  (38436.593-47146.049) | 1595.796  (1463.001-1752.147) | 39 | 173263.321  (156474.553-189309.837) | 2709.069  (2467.268-2935.333) | 40 | 1.638  (1.607-1.668) |
| Cabo Verde | 6560.804  (5971.291-7153.398) | 2774.377  (2532.684-3031.320) | 12 | 34160.012  (31287.689-37044.804) | 6813.398  (6271.198-7393.072) | 7 | 3.138  (2.983-3.293) |
| Cameroon | 119397.885  (107007.260-130696.317) | 2251.969  (2023.678-2456.998) | 26 | 839526.184  (766209.416-917448.068) | 4857.845  (4489.830-5264.069) | 22 | 2.572  (2.463-2.682) |
| Central African Republic | 49777.636  (44799.256-54822.913) | 3491.252  (3165.890-3823.530) | 4 | 220553.480  (200394.657-240753.494) | 6896.734  (6300.021-7480.037) | 6 | 2.282  (2.212-2.352) |
| Chad | 72263.640  (65292.949-79492.241) | 2242.275  (2038.772-2450.719) | 27 | 375720.109  (342547.117-409783.920) | 4739.720  (4323.069-5156.647) | 23 | 2.379  (2.214-2.543) |
| Comoros | 4717.491  (4280.471-5184.534) | 2136.945  (1951.719-2344.259) | 30 | 23314.597  (21531.894-25327.004) | 4177.771  (3856.483-4539.230) | 31 | 2.229  (2.195-2.262) |
| Congo | 32454.135  (29044.192-35684.008) | 2598.533  (2348.414-2849.503) | 15 | 195317.385  (177084.301-213978.273) | 5511.535  (5057.738-5989.321) | 17 | 2.476  (2.434-2.518) |
| Côte d'Ivoire | 142808.591  (128088.837-157133.248) | 2584.060  (2324.330-2814.465) | 17 | 832653.144  (756597.637-902930.395) | 5351.855  (4901.177-5801.916) | 18 | 2.395  (2.330-2.459) |
| Democratic Republic of the Congo | 447437.230  (403661.262-495429.974) | 2398.027  (2184.057-2633.344) | 20 | 2302575.127  (2092791.290-2517221.817) | 4600.867  (4203.761-4996.337) | 27 | 2.076  (2.034-2.118) |
| Equatorial Guinea | 6196.896  (5614.252-6793.289) | 2746.156  (2502.290-3006.767) | 13 | 47595.173  (43346.321-52231.644) | 6209.146  (5659.034-6725.563) | 10 | 2.824  (2.760-2.888) |
| Eritrea | 27192.658  (24060.538-30498.861) | 1905.905  (1727.922-2092.205) | 35 | 146044.533  (132362.918-161375.457) | 3824.237  (3494.107-4142.952) | 34 | 2.293  (2.263-2.323) |
| Eswatini | 8772.066  (7898.969-9758.698) | 2643.429  (2406.861-2938.476) | 14 | 39873.304  (36736.071-43445.973) | 6136.932  (5662.015-6668.018) | 11 | 2.929  (2.804-3.053) |
| Ethiopia | 533664.418  (474221.410-593237.264) | 2261.349  (2016.813-2498.653) | 25 | 1775219.503  (1582274.067-1963889.271) | 3132.183  (2813.379-3463.582) | 38 | 0.875  (0.813-0.937) |
| Gabon | 19119.567  (17415.688-20929.935) | 3083.809  (2824.378-3375.613) | 8 | 83415.488  (76812.029-91274.371) | 6591.485  (6105.588-7121.755) | 8 | 2.508  (2.424-2.592) |
| Gambia | 10405.532  (9274.240-11518.198) | 2274.662  (2065.412-2488.800) | 24 | 65660.572  (60032.179-71789.740) | 5057.562  (4661.383-5488.937) | 20 | 2.661  (2.604-2.718) |
| Ghana | 189541.974  (171213.118-209481.903) | 2416.299  (2190.163-2636.555) | 19 | 1126966.505  (1017344.137-1222855.510) | 5174.078  (4728.819-5615.483) | 19 | 2.503  (2.364-2.641) |
| Guinea | 74880.901  (67878.528-81902.520) | 2065.718  (1876.619-2268.468) | 32 | 305678.370  (280103.089-331187.847) | 4241.896  (3906.548-4601.411) | 30 | 2.190  (2.116-2.265) |
| Guinea-Bissau | 14181.128  (12738.659-15644.646) | 2853.103  (2572.457-3120.798) | 9 | 61090.358  (56073.613-66760.158) | 5767.934  (5317.730-6274.126) | 13 | 2.285  (2.226-2.344) |
| Kenya | 116850.257  (102888.550-130221.998) | 1222.477  (1091.874-1350.519) | 45 | 509747.768  (454635.357-559720.318) | 1858.492  (1674.312-2051.779) | 47 | 1.269  (1.209-1.328) |
| Lesotho | 14541.398  (12982.173-16294.220) | 1633.154  (1461.611-1821.895) | 38 | 56158.167  (51484.711-61242.476) | 4646.604  (4274.961-5048.623) | 25 | 3.673  (3.585-3.762) |
| Liberia | 34441.242  (31257.702-37715.676) | 2554.585  (2345.319-2797.684) | 18 | 174581.599  (157860.278-192967.032) | 5637.960  (5144.934-6125.503) | 16 | 2.680  (2.609-2.750) |
| Madagascar | 81523.297  (73168.710-90797.304) | 1411.715  (1284.074-1553.578) | 40 | 379425.209  (343883.168-418811.595) | 2561.872  (2343.930-2797.724) | 43 | 1.902  (1.881-1.922) |
| Malawi | 49025.107  (43549.103-54916.433) | 1184.729  (1079.717-1299.399) | 46 | 171251.992  (155279.997-187645.096) | 1883.112  (1723.311-2041.561) | 46 | 1.375  (1.311-1.438) |
| Mali | 195477.846  (177952.341-214437.877) | 4182.298  (3814.855-4576.127) | 2 | 1021548.439  (929190.775-1117226.416) | 8614.770  (7885.396-9398.325) | 4 | 2.464  (2.318-2.611) |
| Mauritania | 22028.636  (20056.938-24265.294) | 1930.588  (1761.203-2108.659) | 34 | 84590.364  (78864.216-90256.632) | 3278.896  (3065.128-3466.378) | 37 | 1.581  (1.429-1.733) |
| Mauritius | 39460.296  (36716.153-42916.090) | 5045.519  (4707.759-5453.827) | 1 | 196803.054  (182182.935-211339.881) | 10869.271  (10113.975-11631.117) | 1 | 2.550  (2.414-2.686) |
| Mozambique | 83555.850  (74693.343-92807.982) | 1318.790  (1192.968-1440.622) | 44 | 437079.478  (393607.208-482393.202) | 3037.942  (2759.899-3320.846) | 39 | 2.854  (2.809-2.898) |
| Namibia | 15552.676  (13905.754-17359.778) | 2177.848  (1966.129-2416.040) | 28 | 62219.317  (56236.923-68591.297) | 3903.261  (3543.814-4298.504) | 33 | 1.913  (1.883-1.943) |
| Niger | 84977.312  (76892.528-93428.042) | 2353.288  (2142.024-2568.596) | 21 | 523259.553  (475398.747-573035.933) | 4713.618  (4303.935-5169.239) | 24 | 2.266  (2.236-2.296) |
| Nigeria | 1042967.173  (929113.383-1149834.985) | 2067.062  (1860.136-2284.338) | 31 | 4108042.327  (3684496.466-4516682.710) | 3529.254  (3212.276-3869.788) | 35 | 1.696  (1.659-1.733) |
| Rwanda | 43439.342  (38799.121-48253.088) | 1370.779  (1243.454-1500.896) | 43 | 155724.822  (140368.204-171670.981) | 2083.289  (1902.833-2274.373) | 45 | 1.118  (1.007-1.229) |
| Sao Tome and Principe | 1822.668  (1650.631-2008.259) | 2590.476  (2356.219-2840.112) | 16 | 8276.364  (7609.249-9070.801) | 5674.332  (5219.923-6169.285) | 14 | 2.577  (2.561-2.594) |
| Senegal | 133331.919  (121041.473-146138.784) | 3460.027  (3151.216-3772.890) | 5 | 653534.218  (607554.569-700136.030) | 6971.698  (6508.813-7466.744) | 5 | 2.458  (2.323-2.592) |
| Seychelles | 1929.207  (1755.546-2125.955) | 3341.557  (3043.181-3689.036) | 6 | 12961.095  (11939.029-14024.248) | 10804.438  (10024.551-11618.825) | 2 | 3.821  (3.624-4.018) |
| Sierra Leone | 55230.139  (49822.470-60917.347) | 2307.081  (2080.822-2546.984) | 23 | 250364.396  (227769.444-274434.323) | 4939.599  (4498.098-5342.354) | 21 | 2.537  (2.479-2.594) |
| South Africa | 664018.553  (594636.122-734657.222) | 2846.430  (2548.255-3175.456) | 10 | 2816759.498  (2564649.017-3112713.984) | 5643.490  (5138.410-6233.715) | 15 | 2.329  (2.289-2.370) |
| South Sudan | 39345.845  (34853.548-43876.551) | 1396.746  (1261.103-1548.609) | 41 | 126189.692  (114662.231-138767.261) | 2649.664  (2430.272-2878.051) | 41 | 2.072  (2.054-2.089) |
| Togo | 28432.951  (25459.846-31449.103) | 1761.330  (1592.292-1927.749) | 37 | 170479.265  (156811.830-184670.434) | 3432.423  (3191.777-3686.788) | 36 | 2.157  (2.119-2.194) |
| Uganda | 98910.870  (87940.024-109762.062) | 1375.986  (1242.554-1514.469) | 42 | 494852.761  (446768.638-545012.762) | 2604.723  (2367.665-2844.168) | 42 | 2.011  (1.961-2.062) |
| United Republic of Tanzania | 129558.428  (113905.257-144805.711) | 1124.352  (1011.081-1235.505) | 47 | 724566.972  (661091.142-790159.789) | 2382.032  (2195.931-2581.643) | 44 | 2.471  (2.425-2.517) |
| Zambia | 79274.159  (71693.410-86781.977) | 2316.874  (2127.911-2525.436) | 22 | 437014.669  (397099.377-481908.305) | 4391.802  (4038.971-4786.209) | 28 | 2.073  (2.046-2.099) |
| Zimbabwe | 91466.667  (81649.506-100562.214) | 1939.558  (1746.478-2125.389) | 33 | 350691.840  (322281.703-383337.885) | 4164.288  (3836.354-4553.491) | 32 | 2.648  (2.598-2.699) |

**Table S6 | Deaths Cases and Rates of Diabetes mellitus in African countries and estimated annual percentage changes in 1990 and 2021, along with the rankings of African countries based on age-standardized deaths rates in 1990 and 2021**

| Deaths from diabetes mellitus(1990-2021) in the African region | | | | | | | |
| --- | --- | --- | --- | --- | --- | --- | --- |
| location | 1990 | | | 2021 | | | EAPC 95%CI |
|  | Number(95%UI) | ASR(95%UI) | ASR Ranking | Number(95%UI) | ASR(95%UI) | ASR Ranking |  |
| Algeria | 1203.665  (972.898-1562.211) | 12.683  (10.432-16.254) | 46 | 5481.119  (4389.693-6722.831) | 18.858  (15.233-22.943) | 46 | 1.795  (1.594-1.997) |
| Angola | 1688.764  (1333.523-2167.499) | 49.183  (39.827-61.534) | 14 | 4885.908  (3700.045-6186.323) | 48.895  (38.256-60.218) | 18 | -0.175  (-0.272 to -0.078) |
| Benin | 532.693  (449.160-628.292) | 28.160  (23.798-33.020) | 36 | 1661.283  (1326.658-2050.873) | 36.602  (29.929-44.425) | 37 | 0.838  (0.665-1.012) |
| Botswana | 279.192  (211.956-361.592) | 57.732  (44.903-73.556) | 8 | 733.994  (595.933-900.555) | 60.178  (49.606-73.343) | 11 | 0.426  (0.124-0.728) |
| Burkina Faso | 1342.327  (1130.656-1623.035) | 35.710  (30.205-42.721) | 27 | 2797.939  (2234.461-3452.322) | 34.139  (27.920-41.245) | 42 | -0.140  (-0.212 to -0.068) |
| Burundi | 1157.150  (872.770-1501.626) | 53.095  (40.089-69.248) | 11 | 1869.085  (1329.639-2747.703) | 45.096  (31.762-65.933) | 24 | -0.990  (-1.160 to -0.819) |
| Cabo Verde | 26.799  (22.557-31.368) | 11.248  (9.476-13.094) | 47 | 149.534  (121.552-179.966) | 35.806  (29.027-43.030) | 39 | 3.176  (2.659-3.695) |
| Cameroon | 1507.144  (1192.667-1870.711) | 38.913  (31.043-47.905) | 24 | 5510.431  (4124.229-7317.852) | 51.029  (39.182-66.592) | 15 | 0.773  (0.528-1.018) |
| Central African Republic | 641.717  (539.095-750.493) | 62.470  (52.461-72.048) | 5 | 1214.630  (908.825-1575.206) | 62.887  (48.698-78.509) | 10 | 0.021  (-0.064-0.105) |
| Chad | 618.983  (482.829-806.859) | 23.445  (18.221-30.638) | 42 | 1866.967  (1415.440-2475.559) | 36.210  (27.803-47.264) | 38 | 1.378  (1.075-1.683) |
| Comoros | 77.592  (57.863-101.345) | 44.259  (33.255-57.626) | 19 | 187.928  (129.517-246.178) | 43.874  (30.761-57.100) | 26 | -0.192  (-0.295 to -0.088) |
| Congo | 628.919  (512.203-754.576) | 66.780  (54.960-79.663) | 3 | 1472.496  (1155.595-1883.702) | 64.281  (52.355-79.458) | 9 | -0.335  (-0.451 to -0.218) |
| Côte d'Ivoire | 1088.141  (901.749-1299.855) | 32.512  (27.352-38.512) | 31 | 3911.358  (3009.277-5035.907) | 41.983  (33.413-52.874) | 30 | 0.754  (0.504-1.005) |
| Democratic Republic of the Congo | 6695.623  (5282.146-8327.084) | 50.683  (40.723-62.082) | 12 | 15699.977  (11985.799-20407.687) | 50.494  (38.703-64.648) | 16 | -0.060  (-0.170-0.051) |
| Equatorial Guinea | 98.562  (77.021-120.291) | 56.226  (44.449-67.428) | 9 | 283.811  (199.982-387.883) | 65.067  (47.649-86.623) | 8 | 0.546  (0.270-0.822) |
| Eritrea | 502.067  (390.947-645.967) | 50.332  (39.097-63.744) | 13 | 1257.778  (930.209-1646.702) | 55.365  (42.498-71.039) | 13 | 0.312  (0.231-0.394) |
| Eswatini | 200.370  (160.368-242.369) | 80.448  (65.071-98.028) | 1 | 591.352  (436.706-794.296) | 119.678  (90.920-154.886) | 1 | 1.725  (1.046-2.408) |
| Ethiopia | 12145.980  (10455.021-14062.133) | 66.988  (58.460-77.061) | 2 | 14506.531  (12357.306-16804.894) | 37.774  (32.314-43.953) | 34 | -2.322  (-2.524 to -2.119) |
| Gabon | 340.427  (273.021-435.217) | 64.821  (51.330-82.919) | 4 | 680.349  (511.681-884.755) | 75.123  (57.591-96.816) | 5 | 0.408  (0.261-0.555) |
| Gambia | 84.981  (64.119-108.007) | 27.550  (21.320-34.565) | 37 | 382.098  (287.695-491.028) | 44.104  (33.403-56.146) | 25 | 1.446  (1.298-1.594) |
| Ghana | 1471.350  (1200.780-1835.409) | 27.391  (22.624-33.513) | 39 | 6807.665  (5312.613-8420.884) | 47.747  (37.775-58.625) | 20 | 2.340  (1.997-2.685) |
| Guinea | 897.607  (666.499-1159.819) | 29.207  (21.590-37.626) | 35 | 2080.854  (1596.198-2654.405) | 41.072  (32.056-51.715) | 31 | 1.229  (1.012-1.447) |
| Guinea-Bissau | 156.308  (123.791-189.187) | 42.666  (34.107-51.720) | 21 | 333.781  (261.290-405.286) | 54.402  (43.661-64.746) | 14 | 0.869  (0.702-1.037) |
| Kenya | 2005.696  (1606.446-2493.255) | 27.516  (21.980-34.001) | 38 | 7182.589  (5887.416-8747.537) | 37.261  (30.630-45.256) | 36 | 1.175  (1.058-1.293) |
| Lesotho | 355.287  (290.854-446.535) | 45.090  (37.066-57.118) | 17 | 956.675  (722.258-1210.060) | 97.379  (74.768-121.529) | 3 | 3.442  (2.883-4.005) |
| Liberia | 327.750  (264.242-394.528) | 31.541  (26.138-37.705) | 33 | 795.483  (565.945-1090.519) | 43.392  (31.641-58.323) | 27 | 1.112  (0.965-1.258) |
| Madagascar | 1634.779  (1338.614-1994.128) | 35.237  (28.604-43.248) | 29 | 3333.161  (2472.853-4399.480) | 35.494  (26.534-46.621) | 41 | 0.015  (-0.038-0.068) |
| Malawi | 1549.752  (1297.691-1839.007) | 44.788  (37.347-52.789) | 18 | 3163.833  (2568.759-3779.183) | 48.019  (39.513-56.819) | 19 | -0.019  (-0.276-0.238) |
| Mali | 1146.584  (957.105-1391.675) | 33.450  (28.127-40.178) | 30 | 3160.370  (2484.264-3994.023) | 42.216  (34.018-52.347) | 29 | 0.855  (0.764-0.945) |
| Mauritania | 275.213  (218.144-335.121) | 30.610  (24.391-37.241) | 34 | 701.325  (515.022-943.647) | 37.335  (27.678-50.102) | 35 | 0.413  (0.321-0.506) |
| Mauritius | 384.977  (366.216-404.470) | 53.243  (50.540-55.920) | 10 | 1926.886  (1803.503-2019.903) | 106.474  (99.369-111.635) | 2 | 3.799  (2.822-4.784) |
| Mozambique | 2127.355  (1804.784-2549.168) | 39.361  (34.042-46.273) | 23 | 5122.470  (3876.201-6353.715) | 50.343  (38.613-62.040) | 17 | 1.295  (1.122-1.469) |
| Namibia | 338.290  (283.997-415.875) | 59.421  (49.846-72.052) | 6 | 856.382  (651.704-1088.460) | 72.236  (55.810-90.238) | 6 | 0.409  (0.082-0.737) |
| Niger | 554.216  (420.682-709.744) | 22.190  (16.713-28.451) | 43 | 1930.091  (1415.464-2587.531) | 27.716  (20.679-36.696) | 44 | 0.744  (0.631-0.857) |
| Nigeria | 13884.541  (11488.452-16640.689) | 35.441  (29.592-41.692) | 28 | 28759.503  (23253.208-35557.669) | 37.850  (31.290-45.183) | 33 | 0.151  (0.101-0.201) |
| Rwanda | 1506.050  (1101.877-1994.727) | 58.389  (42.719-77.294) | 7 | 2230.844  (1390.462-3209.643) | 42.436  (27.146-59.504) | 28 | -1.954  (-2.306 to -1.601) |
| Sao Tome and Principe | 8.224  (7.109-9.462) | 13.195  (11.453-15.135) | 45 | 17.916  (14.861-21.496) | 18.430  (15.613-21.671) | 47 | 1.170  (1.036-1.304) |
| Senegal | 925.622  (765.602-1106.154) | 31.619  (26.382-37.739) | 32 | 3106.870  (2398.944-3974.127) | 46.268  (36.143-58.680) | 23 | 1.228  (1.095-1.362) |
| Seychelles | 9.487  (8.450-10.527) | 16.772  (14.938-18.574) | 44 | 26.221  (22.852-30.076) | 24.542  (21.376-28.159) | 45 | 1.585  (1.370-1.801) |
| Sierra Leone | 466.785  (373.232-567.440) | 24.574  (19.883-29.875) | 41 | 1106.612  (853.332-1442.976) | 33.059  (26.074-42.658) | 43 | 1.137  (0.894-1.381) |
| South Africa | 8351.786  (7541.598-9105.940) | 43.140  (38.836-47.176) | 20 | 32423.784  (30132.373-34699.639) | 77.749  (72.202-83.270) | 4 | 2.226  (1.735-2.719) |
| South Sudan | 1103.724  (804.423-1473.825) | 46.770  (34.043-62.165) | 16 | 1928.367  (1419.161-2616.575) | 57.239  (42.667-77.190) | 12 | 0.569  (0.433-0.705) |
| Togo | 274.500  (224.471-334.137) | 24.877  (20.176-30.583) | 40 | 1129.722  (837.995-1523.085) | 35.771  (27.674-46.394) | 40 | 1.143  (0.939-1.347) |
| Uganda | 2386.957  (1672.829-3749.477) | 41.724  (29.206-64.917) | 22 | 5974.053  (4225.890-8654.900) | 46.905  (33.672-65.655) | 22 | -0.037  (-0.271-0.198) |
| United Republic of Tanzania | 3715.842  (3156.551-4344.287) | 37.835  (32.246-43.846) | 25 | 8730.463  (6925.359-11101.898) | 38.273  (30.781-48.174) | 32 | -0.152  (-0.248 to -0.055) |
| Zambia | 1277.425  (1084.992-1521.001) | 48.819  (41.189-58.452) | 15 | 2893.207  (2161.645-3728.608) | 47.003  (36.414-58.919) | 21 | -0.561  (-0.780 to -0.342) |
| Zimbabwe | 1304.291  (1072.616-1549.924) | 37.302  (30.748-44.161) | 26 | 3973.916  (3093.577-5003.032) | 66.128  (52.017-81.930) | 7 | 2.493  (1.874-3.116) |

**Table S7 | Deaths Cases and Rates of Type 1 diabetes mellitus in African countries and estimated annual percentage changes in 1990 and 2021, along with the rankings of African countries based on age-standardized deaths rates in 1990 and 2021**

| Deaths from Type 1 diabetes(1990-2021)in Africa | | | | | | | |
| --- | --- | --- | --- | --- | --- | --- | --- |
| location | 1990 | | | 2021 | | | EAPC 95%CI |
|  | Number(95%UI) | ASR(95%UI) | ASR Ranking | Number(95%UI) | ASR(95%UI) | ASR Ranking |  |
| Algeria | 102.808  (69.448-162.284) | 0.440  (0.304-0.684) | 43 | 138.782  (102.610-197.891) | 0.317  (0.232-0.460) | 47 | -0.911  (-0.976 to -0.845) |
| Angola | 92.294  (64.444-124.076) | 0.999  (0.727-1.361) | 16 | 183.707  (128.298-250.234) | 0.698  (0.467-0.977) | 23 | -1.111  (-1.202 to -1.021) |
| Benin | 36.027  (26.432-48.769) | 0.806  (0.594-1.046) | 28 | 70.598  (52.052-96.914) | 0.598  (0.412-0.837) | 32 | -0.861  (-1.005 to -0.717) |
| Botswana | 5.898  (4.095-8.130) | 0.482  (0.329-0.684) | 41 | 7.963  (5.359-11.150) | 0.330  (0.222-0.451) | 46 | -1.066  (-1.280 to -0.852) |
| Burkina Faso | 85.580  (63.397-109.866) | 1.069  (0.818-1.360) | 14 | 149.153  (110.094-191.469) | 0.733  (0.555-0.959) | 19 | -0.979  (-1.291 to -0.665) |
| Burundi | 67.982  (49.991-86.824) | 1.258  (0.919-1.632) | 5 | 107.143  (74.344-146.131) | 0.922  (0.596-1.302) | 7 | -1.115  (-1.193 to -1.038) |
| Cabo Verde | 1.267  (0.905-1.708) | 0.427  (0.288-0.594) | 45 | 1.746  (1.170-2.430) | 0.333  (0.222-0.458) | 45 | -1.038  (-1.154 to -0.922) |
| Cameroon | 78.363  (59.365-97.291) | 0.922  (0.643-1.256) | 22 | 181.550  (127.824-256.006) | 0.703  (0.469-1.041) | 21 | -0.726  (-0.823 to -0.630) |
| Central African Republic | 29.593  (22.972-38.733) | 1.247  (0.908-1.645) | 7 | 51.625  (36.412-73.957) | 1.095  (0.750-1.548) | 4 | -0.443  (-0.499 to -0.387) |
| Chad | 40.873  (28.166-56.235) | 0.740  0.518-0.979) | 32 | 114.314  (78.612-155.968) | 0.738  (0.532-1.018) | 18 | 0.182  (0.096-0.269) |
| Comoros | 4.874  (3.370-6.263) | 1.070  (0.748-1.463) | 13 | 6.101  (3.999-8.736) | 0.865  (0.542-1.273) | 10 | -1.029  (-1.367 to -0.689) |
| Congo | 21.276  (16.015-27.606) | 1.079  (0.782-1.492) | 12 | 36.540  (25.712-52.216) | 0.767  (0.541-1.098) | 15 | -1.251  (-1.413 to -1.088) |
| Côte d'Ivoire | 77.783  (61.597-101.288) | 0.783  (0.561-1.030) | 30 | 148.924  105.752-199.012) | 0.629  (0.448-0.850) | 28 | -0.541  (-0.674 to -0.408) |
| Democratic Republic of the Congo | 303.598  (236.277-389.334) | 0.926  (0.662-1.259) | 21 | 553.447  (409.017-750.914) | 0.752  (0.524-1.016) | 17 | -0.542  (-0.615 to -0.469) |
| Equatorial Guinea | 3.952  (2.961-5.394) | 1.097  (0.750-1.537) | 11 | 7.794  (4.588-12.151) | 0.606  (0.367-0.906) | 31 | -2.209  (-2.461 to -1.956) |
| Eritrea | 38.274  (26.980-53.272) | 1.250  (0.890-1.716) | 6 | 68.946  (43.276-100.571) | 1.137  (0.709-1.650) | 2 | -0.270  (-0.379 to -0.162) |
| Eswatini | 3.840  (2.848-4.932) | 0.523  (0.389-0.674) | 38 | 6.588  (3.570-9.641) | 0.586  (0.312-0.870) | 34 | 0.794  (0.304-1.286) |
| Ethiopia | 828.601  (648.764-1136.584) | 1.727  (1.312-2.559) | 2 | 770.532  (573.361-996.292) | 0.776  (0.547-1.049) | 13 | -2.957  (-3.111 to -2.803) |
| Gabon | 7.580  (5.512-9.921) | 0.931  (0.635-1.286) | 19 | 10.735  (7.172-15.442) | 0.672  (0.453-0.971) | 24 | -1.081  (-1.236 to -0.926) |
| Gambia | 5.878  (4.397-7.488) | 0.710  (0.512-0.919) | 35 | 13.170  (9.779-17.497) | 0.664  (0.469-0.911) | 25 | -0.335  (-0.568 to -0.100) |
| Ghana | 70.061  (54.504-87.876) | 0.514  (0.387-0.658) | 40 | 140.820  (99.198-185.418) | 0.476  (0.324-0.636) | 39 | 0.034  (-0.113-0.182) |
| Guinea | 51.686  (36.532-70.582) | 0.895  (0.635-1.253) | 23 | 82.169  (60.315-110.750) | 0.725  (0.502-1.004) | 20 | -0.393  (-0.506 to -0.280) |
| Guinea-Bissau | 11.055  (8.125-14.435) | 1.296  (0.976-1.780) | 4 | 15.599  (11.479-20.660) | 0.950  (0.691-1.310) | 6 | -0.811  (-0.902 to -0.720) |
| Kenya | 106.846  (76.472-141.208) | 0.522  (0.362-0.683) | 39 | 221.886  (164.663-288.625) | 0.510  (0.370-0.688) | 38 | 0.293  (0.081-0.505) |
| Lesotho | 5.669  (4.113-7.727) | 0.375  (0.278-0.522) | 46 | 10.638  (6.453-14.964) | 0.579  (0.338-0.846) | 36 | 2.273  (1.862-2.687) |
| Liberia | 20.616  (14.998-27.445) | 0.876  (0.662-1.134) | 25 | 29.311  (20.002-42.255) | 0.632  (0.433-0.931) | 26 | -0.937  (-1.269 to -0.603) |
| Madagascar | 109.499  (84.920-140.897) | 0.930  (0.687-1.199) | 20 | 199.447  (142.932-271.034) | 0.764  (0.532-1.073) | 16 | -0.513  (-0.569--0.458) |
| Malawi | 126.519  (91.705-160.298) | 1.137  (0.917-1.393) | 10 | 153.113  (109.375-200.788) | 0.873  (0.623-1.149) | 8 | -0.866  (-1.007--0.725) |
| Mali | 75.186  (53.802-100.308) | 0.981  (0.757-1.292) | 18 | 134.935  (100.634-180.788) | 0.699  (0.509-0.960) | 22 | -0.880  (-1.044--0.715) |
| Mauritania | 13.044  (10.456-16.225) | 0.758  (0.551-0.969) | 31 | 16.348  (11.506-23.585) | 0.462  (0.308-0.685) | 40 | -1.558  (-1.677 to -1.439) |
| Mauritius | 26.685  (24.921-29.176) | 2.955  (2.737-3.267) | 1 | 40.567  (37.080-43.151) | 2.519  (2.303-2.673) | 1 | -0.366  (-0.556 to -0.176) |
| Mozambique | 170.084  (111.676-235.020) | 1.158  (0.873-1.514) | 9 | 312.358  (211.378-438.977) | 1.096  (0.741-1.536) | 3 | 0.340  (0.154-0.526) |
| Namibia | 5.514  (3.864-7.529) | 0.430  (0.300-0.597) | 44 | 8.612  (5.147-12.976) | 0.370  (0.220-0.557) | 44 | -0.533  (-0.721 to -0.344) |
| Niger | 65.848  (41.125-91.397) | 0.828  (0.606-1.117) | 27 | 124.549  (82.939-176.024) | 0.582  (0.378-0.847) | 35 | -1.124  (-1.293 to -0.954) |
| Nigeria | 666.429  (515.734-848.833) | 0.880  (0.609-1.135) | 24 | 1206.076  (838.611-1610.360) | 0.607  (0.423-0.837) | 30 | -1.028  (-1.193 to -0.862) |
| Rwanda | 99.814  (79.009-125.517) | 1.477  (1.084-2.016) | 3 | 97.137  (61.656-140.823) | 0.802  (0.462-1.238) | 12 | -2.693  (-2.947 to -2.439) |
| Sao Tome and Principe | 0.728  (0.570-0.942) | 0.654  (0.480-0.829) | 36 | 0.857  (0.559-1.260) | 0.518  (0.340-0.743) | 37 | -0.688  (-0.858 to -0.517) |
| Senegal | 56.911  (43.043-73.497) | 0.829  (0.648-1.053) | 26 | 83.352  (61.728-111.578) | 0.629  (0.446-0.882) | 27 | -0.529  (-0.746 to -0.310) |
| Seychelles | 0.373  (0.308-0.485) | 0.608  (0.497-0.802) | 37 | 0.489  (0.317-0.634) | 0.409  (0.267-0.527) | 42 | -0.633  (-0.833 to -0.433) |
| Sierra Leone | 30.223  (20.469-42.733) | 0.739  (0.553-1.014) | 33 | 47.308  (33.827-64.597) | 0.611  (0.430-0.830) | 29 | -0.316  (-0.466 to -0.166) |
| South Africa | 173.983  (144.029-218.797) | 0.477  (0.406-0.590) | 42 | 239.014  (194.712-271.651) | 0.410  (0.335-0.467) | 41 | -0.275  (-0.669-0.121) |
| South Sudan | 58.679  (38.151-81.604) | 0.988  (0.689-1.383) | 17 | 93.954  (63.389-131.133) | 1.017  (0.733-1.434) | 5 | 0.139  (-0.107-0.385) |
| Togo | 22.344  (17.102-29.308) | 0.721  (0.550-0.926) | 34 | 41.996  (29.548-57.005) | 0.598  (0.419-0.835) | 32 | -0.434  (-0.519 to -0.349) |
| Uganda | 133.139  (92.296-183.825) | 0.787  (0.508-1.181) | 29 | 294.309  (206.247-394.202) | 0.773  (0.512-1.107) | 14 | -0.407  (-0.636 to -0.177) |
| United Republic of Tanzania | 303.770  (242.163-370.708) | 1.069  (0.820-1.332) | 14 | 458.001  (322.960-612.416) | 0.809  (0.548-1.103) | 11 | -0.772  (-0.869 to -0.675) |
| Zambia | 103.321  (81.748-128.044) | 1.235  (0.933-1.549) | 8 | 155.991  (104.491-218.491) | 0.871  (0.582-1.261) | 9 | -1.322  (-1.543 to -1.100) |
| Zimbabwe | 20.447  (16.727-25.176) | 0.224  (0.183-0.275) | 47 | 56.308  (40.657-76.292) | 0.381  (0.270-0.533) | 43 | 2.484  (1.981-2.988) |

**Table S8 | Deaths Cases and Rates of Type 2 diabetes mellitus in African countries and estimated annual percentage changes in 1990 and 2021, along with the rankings of African countries based on age-standardized deaths rates in 1990 and 2021**

| Deaths from Type 2 diabetes(1990-2021)in Africa | | | | | | | |
| --- | --- | --- | --- | --- | --- | --- | --- |
| location | 1990 | | | 2021 | | | EAPC 95%CI |
|  | Number(95%UI) | ASR(95%UI) | ASR Ranking | Number(95%UI) | ASR(95%UI) | ASR Ranking |  |
| Algeria | 1100.857  (869.329-1456.474) | 12.243  (9.929-15.856) | 46 | 5342.337  (4274.920-6572.966) | 18.541  (15.015-22.593) | 46 | 1.866  (1.660-2.071) |
| Angola | 1596.470  (1259.920-2042.421) | 48.183  (38.932-60.199) | 14 | 4702.201  (3538.861-5974.124) | 48.197  (37.759-59.418) | 18 | -0.159  (-0.257 to -0.060) |
| Benin | 496.666  (418.785-585.148) | 27.354  (23.102-32.131) | 36 | 1590.685  (1264.592-1969.650) | 36.003  (29.432-43.650) | 37 | 0.875  (0.697-1.055) |
| Botswana | 273.294  (207.221-354.443) | 57.250  (44.502-72.916) | 7 | 726.031  (589.011-889.363) | 59.848  (49.276-72.967) | 11 | 0.436  (0.133-0.739) |
| Burkina Faso | 1256.748  (1042.165-1514.435) | 34.641  (29.216-41.282) | 27 | 2648.786  (2098.835-3290.112) | 33.405  (27.262-40.393) | 42 | -0.119  (-0.192 to -0.046) |
| Burundi | 1089.168  (815.111-1425.852) | 51.837  (39.003-68.060) | 10 | 1761.942  (1240.939-2628.226) | 44.174  (31.070-64.845) | 24 | -0.987  (-1.161 to -0.813) |
| Cabo Verde | 25.532  (21.447-30.034) | 10.821  (9.132-12.669) | 47 | 147.789  (119.921-177.419) | 35.473  (28.774-42.606) | 38 | 3.259  (2.728-3.792) |
| Cameroon | 1428.781  (1131.665-1789.322) | 37.991  (30.259-47.089) | 24 | 5328.881  (3985.856-7060.711) | 50.325  (38.651-65.544) | 15 | 0.800  (0.551-1.050) |
| Central African Republic | 612.124  (510.376-717.141) | 61.223  (51.501-70.683) | 5 | 1163.005  (869.224-1511.083) | 61.792  (47.910-77.200) | 10 | 0.029  (-0.056-0.115) |
| Chad | 578.109  (450.073-760.262) | 22.705  (17.607-29.761) | 42 | 1752.653  (1327.712-2337.552) | 35.472  (27.262-46.376) | 39 | 1.408  (1.098-1.719) |
| Comoros | 72.718  (53.818-96.047) | 43.189  (32.422-56.151) | 19 | 181.827  (125.408-239.389) | 43.009  (30.120-55.953) | 26 | -0.173  (-0.273 to -0.074) |
| Congo | 607.643  (494.513-730.178) | 65.700  (54.018-78.536) | 2 | 1435.956  (1125.227-1834.304) | 63.514  (51.803-78.478) | 9 | -0.322  (-0.438 to -0.206) |
| Côte d'Ivoire | 1010.358  (834.839-1215.682) | 31.729  (26.608-37.670) | 31 | 3762.433  (2899.162-4842.321) | 41.354  (33.015-52.193) | 30 | 0.779  (0.524-1.035) |
| Democratic Republic of the Congo | 6392.025  (5019.041-7998.364) | 49.758  (39.845-61.056) | 12 | 15146.530  (11477.664-19734.128) | 49.742  (37.982-63.697) | 16 | -0.051  (-0.164-0.061) |
| Equatorial Guinea | 94.609  (73.727-115.578) | 55.129  (43.467-65.964) | 9 | 276.017  (194.224-375.702) | 64.461  (47.280-85.674) | 8 | 0.585  (0.309-0.861) |
| Eritrea | 463.793  (358.565-597.865) | 49.082  (38.228-62.225) | 13 | 1188.832  (880.346-1548.505) | 54.227  (41.628-69.474) | 13 | 0.326  (0.245-0.407) |
| Eswatini | 196.530  (156.338-237.933) | 79.925  (64.612-97.470) | 1 | 584.764  (432.669-786.886) | 119.093  (90.487-154.287) | 1 | 1.730  (1.050-2.415) |
| Ethiopia | 11317.378  (9659.733-13181.187) | 65.262  (56.836-75.187) | 3 | 13735.999  (11652.721-16047.110) | 36.999  (31.623-43.076) | 34 | -2.306  (-2.509--2.103) |
| Gabon | 332.847  (266.368-426.391) | 63.889  (50.654-81.859) | 4 | 669.614  (503.162-870.570) | 74.451  (57.075-95.841) | 5 | 0.426  (0.279-0.573) |
| Gambia | 79.103  (59.459-101.038) | 26.840  (20.801-33.682) | 39 | 368.928  (276.571-473.372) | 43.439  (32.839-55.241) | 25 | 1.481  (1.331-1.632) |
| Ghana | 1401.289  (1143.238-1756.437) | 26.876  (22.207-32.873) | 38 | 6666.846  (5204.419-8260.872) | 47.271  (37.391-58.076) | 19 | 2.373  (2.027-2.721) |
| Guinea | 845.921  (630.698-1096.051) | 28.312  (20.932-36.513) | 35 | 1998.685  (1532.303-2560.192) | 40.347  (31.528-50.807) | 31 | 1.268  (1.045-1.492) |
| Guinea-Bissau | 145.254  (114.268-176.433) | 41.369  (32.952-50.120) | 21 | 318.183  (249.048-385.940) | 53.453  (42.854-63.541) | 14 | 0.909  (0.737-1.081) |
| Kenya | 1898.849  (1521.749-2375.379) | 26.994  (21.606-33.427) | 37 | 6960.703  (5691.977-8486.914) | 36.752  (30.176-44.623) | 36 | 1.190  (1.074-1.306) |
| Lesotho | 349.618  (285.532-439.603) | 44.715  (36.751-56.659) | 17 | 946.036  (712.904-1199.206) | 96.800  (74.382-120.902) | 3 | 3.451  (2.890-4.014) |
| Liberia | 307.134  (245.570-370.737) | 30.665  (25.213-36.685) | 33 | 766.172  (542.102-1048.160) | 42.759  (31.180-57.407) | 27 | 1.153  (1.003-1.304) |
| Madagascar | 1525.279  (1231.207-1873.365) | 34.307  (27.808-42.173) | 29 | 3133.714  (2303.205-4150.257) | 34.730  (25.906-45.788) | 41 | 0.028  (-0.026-0.082) |
| Malawi | 1423.233  (1189.323-1708.204) | 43.651  (36.329-51.460) | 18 | 3010.720  (2444.600-3606.075) | 47.146  (38.794-55.861) | 20 | -0.001  (-0.260-0.260) |
| Mali | 1071.398  (895.522-1297.632) | 32.469  (27.225-39.206) | 30 | 3025.434  (2372.891-3797.917) | 41.517  (33.406-51.398) | 29 | 0.893  (0.798-0.988) |
| Mauritania | 262.170  (206.417-319.438) | 29.852  (23.656-36.346) | 34 | 684.977  (503.328-923.067) | 36.873  (27.363-49.488) | 35 | 0.449  (0.355-0.543) |
| Mauritius | 358.291  (339.925-376.573) | 50.289  (47.636-52.948) | 11 | 1886.319  (1766.883-1976.348) | 103.955  (97.019-108.973) | 2 | 3.963  (2.953-4.983) |
| Mozambique | 1957.271  (1656.914-2357.676) | 38.203  (33.010-44.960) | 23 | 4810.112  (3604.014-5970.867) | 49.247  (37.736-60.716) | 17 | 1.320  (1.147-1.494) |
| Namibia | 332.776  (279.316-408.961) | 58.991  (49.495-71.515) | 6 | 847.769  (644.913-1075.558) | 71.866  (55.519-89.779) | 6 | 0.415  (0.087-0.744) |
| Niger | 488.368  (372.061-629.737) | 21.362  (16.009-27.359) | 43 | 1805.542  (1306.301-2429.247) | 27.133  (20.263-35.964) | 44 | 0.797  (0.678-0.917) |
| Nigeria | 13218.112  (10888.565-15916.768) | 34.561  (28.797-40.805) | 28 | 27553.428  (22318.805-33872.547) | 37.243  (30.808-44.459) | 33 | 0.175  (0.122-0.228) |
| Rwanda | 1406.236  (1013.310-1881.995) | 56.912  (41.342-75.323) | 8 | 2133.707  (1328.770-3088.648) | 41.634  (26.630-58.532) | 28 | -1.937  (-2.292 to -1.582) |
| Sao Tome and Principe | 7.496  (6.433-8.661) | 12.542  (10.909-14.436) | 45 | 17.058  (14.242-20.408) | 17.912  (15.261-20.980) | 47 | 1.244  (1.109-1.379) |
| Senegal | 868.712  (719.976-1044.453) | 30.790  (25.588-36.856) | 32 | 3023.518  (2328.656-3885.528) | 45.639  (35.582-58.053) | 23 | 1.263  (1.125-1.400) |
| Seychelles | 9.114  (8.103-10.129) | 16.165  (14.381-17.927) | 44 | 25.732  (22.452-29.457) | 24.132  (21.065-27.751) | 45 | 1.640  (1.418-1.862) |
| Sierra Leone | 436.562  (343.400-536.719) | 23.835  (19.245-29.070) | 41 | 1059.304  (815.812-1386.030) | 32.448  (25.563-41.851) | 43 | 1.172  (0.923-1.422) |
| South Africa | 8177.802  (7377.276-8907.856) | 42.663  (38.417-46.648) | 20 | 32184.770  (29911.123-34447.759) | 77.339  (71.798-82.791) | 4 | 2.244  (1.751-2.740) |
| South Sudan | 1045.045  (756.589-1414.038) | 45.782  (33.304-61.065) | 16 | 1834.413  (1352.522-2496.858) | 56.222  (41.935-75.957) | 12 | 0.577  (0.443-0.712) |
| Togo | 252.156  (205.173-310.478) | 24.156  (19.541-29.762) | 40 | 1087.727  (806.283-1460.730) | 35.173  (27.242-45.653) | 40 | 1.179  (0.970-1.388) |
| Uganda | 2253.818  (1566.832-3572.750) | 40.936  (28.619-63.754) | 22 | 5679.744  (4026.868-8305.630) | 46.132  (32.973-64.535) | 21 | -0.030  (-0.265-0.206) |
| United Republic of Tanzania | 3412.072  (2875.679-4012.326) | 36.767  (31.311-42.693) | 26 | 8272.463  (6533.481-10593.841) | 37.464  (30.101-47.330) | 32 | -0.136  (-0.235 to -0.037) |
| Zambia | 1174.105  (983.501-1411.469) | 47.584  (40.203-57.254) | 15 | 2737.216  (2042.540-3518.137) | 46.131  (35.737-57.726) | 22 | -0.545  (-0.764 to -0.324) |
| Zimbabwe | 1283.844  (1054.293-1527.459) | 37.078  (30.551-43.902) | 25 | 3917.608  (3047.445-4931.298) | 65.747  (51.748-81.445) | 7 | 2.493  (1.873-3.117) |

**Table S9 | Dalys Cases and Rates of Diabetes mellitus in African countries and estimated annual percentage changes in 1990 and 2021, along with the rankings of African countries based on age-standardized Dalys rates in 1990 and 2021**

| Dalys from diabetes(1990-2021)in Africa | | | | | | | |
| --- | --- | --- | --- | --- | --- | --- | --- |
| location | 1990 | | | 2021 | | | EAPC 95%CI |
|  | Number(95%UI) | ASR(95%UI) | ASR Ranking | Number(95%UI) | ASR(95%UI) | ASR Ranking |  |
| Algeria | 76076.527  (60714.214-95409.217) | 572.274  (461.607-714.612) | 45 | 433687.207  (328921.387-561425.614) | 1152.389  (882.438-1482.938) | 36 | 2.432  (2.352-2.511) |
| Angola | 66432.035  (54832.189-82709.108) | 1462.313  (1211.260-1809.515) | 11 | 231484.136  (187161.785-286091.512) | 1655.141  (1356.909-2029.674) | 14 | 0.323  (0.250-0.396) |
| Benin | 20344.430  (17257.578-23606.082) | 892.045  (767.332-1035.185) | 34 | 82782.885  (66927.991-102849.285) | 1355.580  (1093.216-1637.718) | 28 | 1.271  (1.185-1.358) |
| Botswana | 8535.656  (6724.552-10820.031) | 1469.547  (1162.169-1849.627) | 10 | 25249.609  (20869.195-30708.480) | 1692.417  (1427.730-2034.349) | 12 | 0.654  (0.417-0.893) |
| Burkina Faso | 46503.990  (39746.133-55220.898) | 978.281  (846.721-1154.524) | 29 | 122909.610  (99997.932-151223.634) | 1142.904  (940.070-1397.076) | 37 | 0.502  (0.412-0.591) |
| Burundi | 37860.069  (30236.326-47253.513) | 1423.936  (1114.678-1798.789) | 12 | 71400.737  (55149.024-98014.990) | 1259.245  (944.185-1720.357) | 33 | -0.829  (-0.991 to -0.667) |
| Cabo Verde | 1204.502  (1007.388-1468.198) | 510.063  (425.916-623.156) | 47 | 6121.625  (4950.853-7603.140) | 1322.318  (1075.901-1645.216) | 29 | 2.880  (2.570-3.191) |
| Cameroon | 52767.041  (43575.805-62666.112) | 1072.691  (884.327-1274.993) | 23 | 224397.326  (171953.653-284343.462) | 1553.983  (1216.945-1973.542) | 19 | 1.098  (0.877-1.319) |
| Central African Republic | 24377.104  (20666.588-28049.629) | 1851.807  (1582.412-2144.758) | 4 | 57421.039  (45537.526-71114.091) | 2127.961  (1715.956-2595.756) | 6 | 0.429  (0.361-0.498) |
| Chad | 23152.524  (18784.637-28151.894) | 736.642  (600.069-892.946) | 40 | 86026.326  (69187.786-106850.328) | 1231.162  (994.106-1514.856) | 34 | 1.619  (1.356-1.883) |
| Comoros | 2780.264  (2181.240-3510.457) | 1239.932  (982.724-1566.125) | 18 | 7092.342  (5395.929-8911.259) | 1366.940  (1041.496-1703.714) | 27 | 0.162  (0.039-0.285) |
| Congo | 21316.788  (17628.739-25098.535) | 1827.726  (1516.866-2140.776) | 5 | 60084.674  (48174.141-74949.478) | 1959.225  (1608.542-2393.597) | 8 | 0.013  (-0.102-0.127) |
| Côte d'Ivoire | 45946.842  (38817.704-54493.332) | 958.964  (819.590-1131.247) | 32 | 180790.400  (145055.771-225200.135) | 1386.823  (1130.587-1715.694) | 26 | 1.111  (0.921-1.302) |
| Democratic Republic of the Congo | 237258.330  (191605.930-287682.683) | 1376.244  (1116.871-1656.770) | 14 | 652555.400  (514438.054-816494.045) | 1554.301  (1240.140-1937.548) | 17 | 0.358  (0.263-0.453) |
| Equatorial Guinea | 3457.915(2771.890-4215.817) | 1608.746  (1303.550-1940.481) | 7 | 12134.670  (9217.291-15703.417) | 1993.631  (1529.406-2548.210) | 7 | 0.756  (0.491-1.023) |
| Eritrea | 20082.821  (16226.697-25634.163) | 1420.291  (1150.970-1766.052) | 13 | 52167.034  (40254.303-66763.415) | 1604.863  (1256.858-2026.013) | 15 | 0.392  (0.337-0.447) |
| Eswatini | 6066.973  (4974.226-7222.573) | 2039.721  (1679.997-2421.834) | 1 | 19365.853  (14872.380-25183.254) | 3295.244  (2589.353-4243.360) | 2 | 1.938  (1.282-2.598) |
| Ethiopia | 439764.075  (379359.403-510118.086) | 1867.019  (1625.061-2143.035) | 3 | 563802.152  (478412.178-652676.367) | 1120.758  (957.735-1290.242) | 38 | -2.103  (-2.303 to -1.902) |
| Gabon | 10457.809  (8505.928-12968.039) | 1768.938  (1437.215-2192.769) | 6 | 25612.932  (20439.707-32174.444) | 2267.388  (1833.993-2843.380) | 4 | 0.758  (0.636-0.881) |
| Gambia | 3398.705  (2732.449-4137.023) | 824.971  (660.549-1010.137) | 38 | 15628.344  (12632.123-19536.275) | 1406.049  (1135.664-1760.577) | 25 | 1.656  (1.528-1.785) |
| Ghana | 59544.434  (49768.688-71572.885) | 834.699  (700.204-1006.841) | 37 | 278678.612  (224171.417-340765.985) | 1500.332  (1214.390-1837.236) | 21 | 2.327  (2.027-2.628) |
| Guinea | 30709.740  (23669.269-37801.096) | 854.288  (667.241-1051.745) | 36 | 81144.696  (65547.841-99434.348) | 1278.432  (1044.265-1575.910) | 31 | 1.346  (1.188-1.505) |
| Guinea-Bissau | 6066.119  (5016.820-7247.896) | 1296.267  (1079.223-1542.731) | 16 | 15234.899  (12348.724-18635.193) | 1743.333  (1425.757-2096.597) | 11 | 1.001  (0.861-1.141) |
| Kenya | 66998.779  (56032.373-80303.274) | 721.073  (607.347-871.871) | 43 | 247028.870  (209838.832-293519.028) | 992.642  (842.550-1180.472) | 46 | 1.201  (1.064-1.337) |
| Lesotho | 9937.004  (8320.011-12042.362) | 1149.156  (965.093-1400.537) | 21 | 29592.596  (22954.063-37169.703) | 2639.981  (2064.403-3273.636) | 3 | 3.539  (3.036-4.045) |
| Liberia | 11964.287  (9858.631-14181.862) | 932.973  (777.461-1121.371) | 33 | 37001.445  (28621.927-47717.603) | 1446.300  (1143.155-1845.447) | 24 | 1.496  (1.383-1.609) |
| Madagascar | 56816.352  (47508.714-67602.860) | 967.569  (805.059-1149.567) | 30 | 140356.418  (111832.591-176493.490) | 1051.987  (834.276-1323.661) | 44 | 0.266  (0.215-0.317) |
| Malawi | 53370.502  (45704.590-61821.406) | 1159.976  (991.026-1356.297) | 20 | 109557.743  (90824.254-130892.385) | 1282.528  (1074.297-1515.219) | 30 | 0.062  (-0.187-0.312) |
| Mali | 50360.691  (42637.631-60124.316) | 1135.609  (966.739-1346.899) | 22 | 171927.669  (138431.996-214815.706) | 1675.504  (1360.718-2077.835) | 13 | 1.353  (1.268-1.437) |
| Mauritania | 9218.044  (7693.016-10858.313) | 856.849  (709.801-1008.300) | 35 | 24257.812  (19246.956-30181.667) | 1059.739  (834.965-1328.171) | 43 | 0.492  (0.417-0.567) |
| Mauritius | 14800.564  (13475.281-16370.117) | 1871.368  (1700.100-2068.790) | 2 | 64764.437  (58615.143-72875.423) | 3514.449  (3183.147-3949.590) | 1 | 3.144  (2.484-3.810) |
| Mozambique | 75128.398  (62838.864-88566.830) | 1066.804  (918.918-1244.394) | 24 | 203963.088  (158878.357-252805.262) | 1498.840  (1178.599-1829.023) | 22 | 1.580  (1.409-1.751) |
| Namibia | 10098.931  (8670.488-12294.693) | 1509.901  (1284.893-1824.100) | 9 | 26618.408  (20931.074-33174.854) | 1903.093  (1513.801-2345.477) | 9 | 0.533  (0.231-0.836) |
| Niger | 25560.058  (20617.688-30924.626) | 728.444  (591.367-878.819) | 41 | 99408.935  (78574.264-126005.229) | 1017.894  (802.616-1292.733) | 45 | 1.091  (1.021-1.160) |
| Nigeria | 457837.013  (385928.110-542170.922) | 963.191  (819.685-1131.500) | 31 | 1114259.323  (902255.447-1363731.993) | 1095.220  (902.382-1326.988) | 40 | 0.323  (0.276-0.369) |
| Rwanda | 51174.339  (39508.976-64703.069) | 1546.364  (1157.658-2006.565) | 8 | 77135.406  (52538.089-104426.630) | 1120.469  (765.887-1525.862) | 39 | -1.979  (-2.337 to -1.620) |
| Sao Tome and Principe | 386.412  (318.719-467.211) | 542.814  (452.160-666.988) | 46 | 1168.453  (934.581-1489.893) | 910.366  (735.778-1149.820) | 47 | 1.664  (1.592-1.737) |
| Senegal | 37565.184  (31621.732-44849.450) | 1027.020  (864.359-1225.689) | 26 | 134828.834  (107279.755-165360.919) | 1604.495  (1272.485-1967.591) | 16 | 1.532  (1.404-1.660) |
| Seychelles | 407.285  (348.797-483.238) | 711.800  (610.110-844.213) | 44 | 1809.924  (1418.922-2306.385) | 1521.571  (1198.644-1925.879) | 20 | 2.629  (2.483-2.776) |
| Sierra Leone | 17359.853  (14155.226-21120.374) | 759.550  (635.913-914.911) | 39 | 51028.496  (40850.548-64419.812) | 1164.914  (944.930-1456.793) | 35 | 1.551  (1.376-1.725) |
| South Africa | 278249.972  (252445.720-309233.003) | 1265.394  (1144.288-1403.413) | 17 | 1033716.792  (935561.109-1144056.410) | 2194.471  (1993.305-2419.610) | 5 | 2.059  (1.689-2.430) |
| South Sudan | 34763.543  (26615.778-44764.830) | 1214.495  (925.324-1575.398) | 19 | 69530.903  (54509.541-90844.284) | 1554.253  (1213.101-2031.648) | 18 | 0.702  (0.539-0.866) |
| Togo | 10878.072  (9181.918-12908.595) | 726.850  (608.526-873.624) | 42 | 46897.673  (36928.172-59533.159) | 1091.683  (880.564-1366.387) | 41 | 1.296  (1.141-1.450) |
| Uganda | 77245.578  (57656.925-113612.692) | 1064.150  (781.447-1586.823) | 25 | 216405.703  (167344.104-292765.721) | 1268.713  (952.614-1716.130) | 32 | 0.117  (-0.110-0.344) |
| United Republic of Tanzania | 126675.631  (109590.615-146635.186) | 987.690  (850.707-1136.237) | 27 | 317240.421  (255813.543-393257.654) | 1089.427  (886.335-1345.621) | 42 | 0.167  (0.094-0.240) |
| Zambia | 47603.311  (40580.832-55425.356) | 1373.323  (1181.527-1608.004) | 15 | 127776.738  (101566.493-158791.645) | 1496.396  (1212.150-1828.042) | 23 | -0.142  (-0.334-0.051) |
| Zimbabwe | 41198.276  (34288.824-48019.182) | 980.826  (816.586-1144.519) | 28 | 138331.979  (111421.323-169829.092) | 1881.206  (1529.285-2284.430) | 10 | 2.670  (2.116-3.228) |

**Table S10 | Dalys Cases and Rates of Type 1 diabetes mellitus in African countries and estimated annual percentage changes in 1990 and 2021, along with the rankings of African countries based on age-standardized Dalys rates in 1990 and 2021**

| Dalys from Type 1 diabetes(1990-2021)in Africa | | | | | | | |
| --- | --- | --- | --- | --- | --- | --- | --- |
| location | 1990 | | | 2021 | | | EAPC 95%CI |
|  | Number(95%UI) | ASR(95%UI) | ASR Ranking | Number(95%UI) | ASR(95%UI) | ASR Ranking |  |
| Algeria | 9180.594  (6610.761-14208.459) | 36.677  (27.105-52.707) | 45 | 13757.901  (10686.162-17707.660) | 30.885  (23.966-39.886) | 46 | -0.491  (-0.555 to -0.428) |
| Angola | 6977.834  (4785.221-9323.833) | 64.837  (48.375-83.987) | 18 | 14188.514  (10587.343-18431.983) | 47.269  (34.747-61.957) | 24 | -0.956  (-1.040 to -0.872) |
| Benin | 2815.317  (2000.210-3808.352) | 53.416  (43.266-67.776) | 29 | 5732.231  (4223.268-7509.879) | 42.365  (32.673-55.003) | 34 | -0.680  (-0.782 to -0.578) |
| Botswana | 548.666  (414.394-698.366) | 44.344  (33.194-57.350) | 39 | 873.076  (648.492-1124.681) | 36.745  (27.488-47.157) | 40 | -0.472  (-0.585 to -0.359) |
| Burkina Faso | 6198.222  (4416.569-8155.119) | 65.247  (52.917-79.701) | 17 | 11668.158  (8648.357-14705.456) | 50.670  (38.837-63.430) | 19 | -0.612  (-0.829 to -0.394) |
| Burundi | 5430.743  (4037.516-6897.992) | 87.322  (68.268-108.440) | 5 | 8667.857  (6427.800-11272.720) | 65.235  (49.310-84.096) | 9 | -0.916  (-1.003 to -0.828) |
| Cabo Verde | 99.522  (78.617-126.096) | 31.057  (24.324-39.058) | 46 | 148.048  (113.195-189.771) | 26.749  (20.635-34.151) | 47 | -0.675  (-0.765 to -0.584) |
| Cameroon | 5745.703  (4485.760-7099.866) | 56.732  (44.950-70.220) | 25 | 13892.502  (10230.664-18118.215) | 46.721  (35.247-62.478) | 25 | -0.527  (-0.606 to -0.448) |
| Central African Republic | 2170.654  (1699.562-2700.114) | 78.610  (63.076-99.568) | 11 | 3774.419  (2747.014-5033.992) | 69.857  (51.380-92.205) | 5 | -0.393  (-0.442 to -0.344) |
| Chad | 3118.157  (2089.257-4290.363) | 48.270  (36.602-62.770) | 33 | 9029.880  (6293.161-12073.543) | 48.454  (36.715-63.240) | 21 | 0.180  (0.102-0.258) |
| Comoros | 402.750  (291.227-512.230) | 77.541  (56.524-97.568) | 12 | 482.645  (361.896-637.532) | 64.556  (47.858-85.697) | 10 | -0.844  (-1.105 to -0.582) |
| Congo | 1550.601  (1238.057-1884.695) | 68.705  (54.248-87.220) | 14 | 2700.421  (1990.731-3627.952) | 51.944  (38.702-69.463) | 17 | -0.987  (-1.124 to -0.849) |
| Côte d'Ivoire | 6081.111  (4789.843-7694.897) | 50.712  (41.393-63.492) | 30 | 11783.673  (8659.467-15084.047) | 43.706  (33.030-55.991) | 29 | -0.362  (-0.479 to -0.244) |
| Democratic Republic of the Congo | 22499.220  (17490.228-27992.705) | 58.448  (46.649-73.517) | 23 | 40780.293  (31202.048-52543.767) | 48.584  (37.364-62.697) | 20 | -0.472  (-0.541 to -0.403) |
| Equatorial Guinea | 281.776  (213.874-366.760) | 67.253  (51.046-88.539) | 16 | 608.514  (409.259-900.643) | 42.814  (29.815-61.212) | 32 | -1.689  (-1.903 to -1.475) |
| Eritrea | 3095.041  (2212.755-4147.372) | 85.886  (64.192-113.442) | 7 | 5393.148  (3819.432-7436.086) | 79.462  (57.267-108.061) | 3 | -0.229  (-0.329 to -0.130) |
| Eswatini | 361.093  (280.687-448.639) | 48.231  (37.901-59.558) | 34 | 592.447  (408.143-795.907) | 52.818  (36.216-70.572) | 16 | 0.548  (0.265-0.833) |
| Ethiopia | 64170.711  (49716.283-81090.962) | 113.365  (91.635-153.430) | 2 | 64951.280  (52462.015-79831.847) | 59.211  (46.395-73.821) | 13 | -2.344  (-2.461 to -2.226) |
| Gabon | 540.953  (421.689-667.329) | 59.846  (45.726-75.012) | 20 | 826.930  (611.095-1127.630) | 47.849  (35.653-64.833) | 23 | -0.729  (-0.842 to -0.616) |
| Gambia | 460.507  (353.506-579.245) | 47.178  (37.195-58.298) | 36 | 1016.334  (799.131-1274.780) | 44.990  (35.027-58.296) | 26 | -0.283  (-0.477 to -0.090) |
| Ghana | 5741.277  (4493.488-7098.966) | 38.322  (31.362-47.011) | 43 | 11461.317  (8880.184-14348.758) | 35.312  (27.219-44.403) | 41 | -0.107  (-0.195 to -0.019) |
| Guinea | 3870.834  (2589.398-5367.316) | 58.512  (44.074-76.666) | 22 | 6359.297  (4745.606-8179.823) | 48.351  (37.070-62.024) | 22 | -0.385  (-0.474 to -0.296) |
| Guinea-Bissau | 812.447  (581.608-1072.520) | 79.751  (62.317-100.567) | 10 | 1157.180  (885.665-1497.439) | 59.797  (45.777-77.827) | 12 | -0.781  (-0.875 to -0.688) |
| Kenya | 9532.889  (7157.569-12253.243) | 41.576  (32.548-53.341) | 40 | 19361.052  (15391.239-23580.644) | 40.659  (31.742-49.939) | 36 | 0.181  (0.044-0.318) |
| Lesotho | 567.307  (439.763-738.042) | 38.165  (29.747-49.144) | 44 | 943.200  (664.177-1219.354) | 51.056  (35.721-66.434) | 18 | 1.384  (1.171-1.597) |
| Liberia | 1569.036  (1076.251-2158.378) | 57.182  (44.889-72.197) | 24 | 2263.590  (1631.352-2995.764) | 42.921  (31.787-57.243) | 31 | -0.868  (-1.163 to -0.572) |
| Madagascar | 9020.842  (6953.858-11465.471) | 67.633  (55.911-83.540) | 15 | 16395.812  (12645.951-21105.112) | 56.691  (44.319-73.068) | 15 | -0.432  (-0.483 to -0.380) |
| Malawi | 10724.938  (7413.503-13746.768) | 85.466  (67.065-103.197) | 8 | 12704.458  (9729.699-16422.409) | 65.267  (49.989-83.499) | 8 | -0.817  (-0.922 to -0.712) |
| Mali | 5510.740  (3765.607-7470.937) | 60.331  (48.039-76.451) | 19 | 10369.151  (7812.581-13418.868) | 44.891  (35.663-57.238) | 27 | -0.796  (-0.900 to -0.692) |
| Mauritania | 954.595  (760.719-1175.112) | 47.674  (38.580-58.804) | 35 | 1331.022  (1017.913-1712.692) | 33.433  (25.093-44.428) | 44 | -1.150  (-1.217 to -1.084) |
| Mauritius | 1368.413  (1244.921-1505.130) | 133.350  (121.678-146.645) | 1 | 1932.811  (1738.935-2112.228) | 128.500  (115.666-140.083) | 1 | 0.070  (-0.118-0.258) |
| Mozambique | 14327.426  (9072.269-19844.659) | 87.203  (64.811-113.942) | 6 | 25501.018  (17785.857-34854.055) | 79.697  (59.443-106.580) | 2 | 0.122  (-0.027-0.272) |
| Namibia | 519.895  (396.275-674.680) | 39.791  (30.213-51.349) | 41 | 858.343  (604.044-1139.113) | 36.801  (26.105-48.667) | 39 | -0.218  (-0.306 to -0.131) |
| Niger | 5221.324  (3159.042-7389.961) | 54.726  (40.913-71.138) | 27 | 10053.838  (6832.432-13674.384) | 39.151  (28.314-52.644) | 38 | -1.090  (-1.232 to -0.948) |
| Nigeria | 48079.904  (37500.226-59783.123) | 54.504  (42.543-67.751) | 28 | 98572.096  (70210.196-126403.134) | 43.989  (32.978-56.847) | 28 | -0.568  (-0.675 to -0.461) |
| Rwanda | 7862.267  (6133.549-9593.842) | 100.419  (81.768-123.192) | 3 | 8070.988  (6044.971-10777.849) | 60.754  (43.993-82.622) | 11 | -2.146  (-2.334 to -1.958) |
| Sao Tome and Principe | 57.070  (42.684-74.409) | 45.089  (36.187-55.690) | 38 | 66.642  (48.951-89.998) | 34.658  (25.183-45.743) | 42 | -0.802  (-0.907 to -0.698) |
| Senegal | 4406.144  (3228.934-5659.093) | 55.286  (44.420-67.304) | 26 | 6463.617  (5081.638-8170.369) | 43.307  (34.002-54.782) | 30 | -0.524  (-0.683 to -0.365) |
| Seychelles | 24.980  (20.518-30.437) | 38.738  (31.855-47.250) | 42 | 37.045  (27.146-46.764) | 30.897  (22.669-38.746) | 45 | -0.309  (-0.441 to -0.177) |
| Sierra Leone | 2361.453  (1523.745-3327.783) | 50.425  (37.736-68.109) | 31 | 3756.228  (2787.116-4976.768) | 42.688  (32.481-55.060) | 33 | -0.368  (-0.473 to -0.263) |
| South Africa | 16155.582  (13371.520-20241.898) | 45.133  (36.982-55.672) | 37 | 23339.498  (18682.686-28446.164) | 40.308  (32.279-49.161) | 37 | -0.225  (-0.454-0.005) |
| South Sudan | 4888.665  (3186.476-6728.277) | 71.961  (52.560-94.813) | 13 | 7675.917  (5184.474-10555.910) | 72.863  (53.690-95.467) | 4 | 0.118  (-0.096-0.333) |
| Togo | 1776.088  (1327.522-2309.170) | 48.577  (39.743-61.183) | 32 | 3290.998  (2433.185-4245.197) | 41.393  (31.263-53.503) | 35 | -0.404  (-0.464 to -0.344) |
| Uganda | 11548.519  (8373.211-15057.972) | 58.646  (44.644-77.330) | 21 | 25141.337  (19262.716-31756.645) | 58.211  (43.936-74.983) | 14 | -0.246  (-0.413 to -0.078) |
| United Republic of Tanzania | 26061.870  (20777.385-32251.801) | 82.593  (69.329-99.240) | 9 | 40201.103  (30345.057-52375.654) | 65.785  (49.854-85.014) | 6 | -0.590  (-0.683 to -0.497) |
| Zambia | 8645.913  (6746.129-10865.002) | 89.892  (73.115-109.716) | 4 | 12928.193  (9347.154-17465.466) | 65.325  (47.913-88.498) | 7 | -1.142  (-1.318 to -0.965) |
| Zimbabwe | 2192.739  (1786.246-2698.453) | 23.856  (19.112-29.857) | 47 | 5090.309  (3924.662-6511.550) | 33.723  (25.863-42.666) | 43 | 1.503  (1.254-1.752) |

**Table S11 | Dalys Cases and Rates of Type 2 diabetes mellitus in African countries and estimated annual percentage changes in 1990 and 2021, along with the rankings of African countries based on age-standardized Dalys rates in 1990 and 2021**

| Dalys from Type 2 diabetes(1990-2021)in Africa | | | | | | | |
| --- | --- | --- | --- | --- | --- | --- | --- |
| location | 1990 | | | 2021 | |  | EAPC 95%CI |
|  | Number(95%UI) | ASR(95%UI) | ASR Ranking | Number(95%UI) | ASR(95%UI) | ASR Ranking |  |
| Algeria | 66895.932  (52939.135-85746.218) | 535.597  (431.582-673.968) | 45 | 419929.306  (317650.974-546949.886) | 1121.504  (858.771-1447.529) | 36 | 2.561  (2.483-2.638) |
| Angola | 59454.201  (48557.420-74013.600) | 1397.476  (1153.545-1725.817) | 11 | 217295.621  (174411.426-269222.927) | 1607.872  (1317.246-1972.571) | 14 | 0.372  (0.297-0.447) |
| Benin | 17529.112  (15003.362-20503.898) | 838.629  (719.225-976.032) | 34 | 77050.654  (61453.394-96109.854) | 1313.215  (1062.038-1590.870) | 27 | 1.361  (1.268-1.454) |
| Botswana | 7986.990  (6257.172-10158.111) | 1425.203  (1126.913-1799.636) | 10 | 24376.533  (20132.653-29714.361) | 1655.672  (1397.348-1995.172) | 12 | 0.684  (0.442-0.925) |
| Burkina Faso | 40305.769  (34053.203-47729.612) | 913.034  (788.122-1079.050) | 28 | 111241.453  (89590.512-137810.801) | 1092.234  (893.050-1337.888) | 37 | 0.565  (0.481-0.649) |
| Burundi | 32429.326  (25058.680-41316.766) | 1336.614  (1038.917-1707.238) | 12 | 62732.881  (46687.227-87611.072) | 1194.010  (885.105-1644.539) | 33 | -0.824  (-0.997 to -0.651) |
| Cabo Verde | 1104.980  (915.631-1368.714) | 479.007  (396.844-592.453) | 47 | 5973.576  (4824.783-7410.386) | 1295.568  (1053.824-1611.499) | 29 | 3.011  (2.685-3.339) |
| Cameroon | 47021.338  (38508.401-56550.196) | 1015.959  (833.076-1215.679) | 23 | 210504.824  (160407.978-268192.590) | 1507.261  (1177.251-1916.285) | 17 | 1.166  (0.936-1.396) |
| Central African Republic | 22206.450  (18757.014-25737.116) | 1773.197  (1510.757-2059.385) | 2 | 53646.620  (42546.152-66805.835) | 2058.104  (1663.661-2515.922) | 6 | 0.461  (0.391-0.532) |
| Chad | 20034.367  (16322.006-24500.959) | 688.371  (560.356-836.636) | 40 | 76996.446  (61730.758-95945.670) | 1182.708  (955.075-1460.810) | 34 | 1.695  (1.420-1.971) |
| Comoros | 2377.514  (1820.076-3032.506) | 1162.390  (918.434-1472.170) | 18 | 6609.697  (5027.985-8300.967) | 1302.384  (994.205-1621.163) | 28 | 0.221  (0.104-0.338) |
| Congo | 19766.187  (16188.378-23357.940) | 1759.021  (1458.776-2056.698) | 3 | 57384.252  (46212.936-71677.150) | 1907.281  (1563.191-2332.620) | 8 | 0.046  (-0.068-0.161) |
| Côte d'Ivoire | 39865.731  (33588.214-47750.399) | 908.252  (771.778-1073.561) | 30 | 169006.727  (134991.024-211090.599) | 1343.117  (1096.365-1660.472) | 26 | 1.174  (0.974-1.374) |
| Democratic Republic of the Congo | 214759.110  (172018.905-261919.815) | 1317.796  (1068.705-1589.456) | 14 | 611775.106  (481699.179-766854.737) | 1505.717  (1196.239-1881.957) | 18 | 0.390  (0.293-0.488) |
| Equatorial Guinea | 3176.138  (2539.124-3877.897) | 1541.493  (1245.527-1852.353) | 7 | 11526.156  (8726.120-14902.898) | 1950.816  (1497.709-2493.025) | 7 | 0.834  (0.569-1.101) |
| Eritrea | 16987.780  (13511.536-21562.793) | 1334.405  (1078.023-1664.844) | 13 | 46773.886  (36231.301-59814.167) | 1525.401  (1194.738-1914.138) | 16 | 0.429  (0.375-0.482) |
| Eswatini | 5705.879  (4634.085-6786.777) | 1991.490  (1635.886-2370.049) | 1 | 18773.406  (14477.265-24504.384) | 3242.426  (2550.143-4173.458) | 2 | 1.965  (1.302-2.632) |
| Ethiopia | 375593.364  (323482.002-439120.404) | 1753.654  (1525.395-2017.614) | 4 | 498850.872  (421662.366-583404.967) | 1061.548  (907.572-1224.141) | 38 | -2.088  (-2.293 to -1.882) |
| Gabon | 9916.857  (8008.923-12339.478) | 1709.093  (1380.965-2124.674) | 6 | 24786.001  (19771.358-31098.080) | 2219.538  (1793.936-2786.211) | 4 | 0.800  (0.676-0.925) |
| Gambia | 2938.197  (2330.400-3643.042) | 777.793  (621.589-955.238) | 38 | 14612.010  (11729.635-18385.226) | 1361.059  (1096.533-1705.637) | 25 | 1.743  (1.612-1.874) |
| Ghana | 53803.157  (44736.772-65394.809) | 796.376  (667.650-966.544) | 36 | 267217.295  (214712.966-326853.736) | 1465.020  (1186.013-1793.847) | 21 | 2.410  (2.101-2.720) |
| Guinea | 26838.906  (20995.352-32732.038) | 795.775  (624.648-977.181) | 37 | 74785.399  (60165.087-92016.523) | 1230.081  (1002.960-1516.737) | 30 | 1.440  (1.271-1.610) |
| Guinea-Bissau | 5253.672  (4316.304-6352.753) | 1216.516  (1005.446-1458.877) | 17 | 14077.719  (11379.535-17332.479) | 1683.537  (1377.715-2026.135) | 11 | 1.088  (0.941-1.234) |
| Kenya | 57465.890  (48176.457-69840.420) | 679.497  (571.184-826.678) | 41 | 227667.818  (192907.769-271991.329) | 951.983  (806.165-1133.667) | 46 | 1.253  (1.116-1.391) |
| Lesotho | 9369.696  (7850.179-11433.972) | 1110.991  (932.372-1356.658) | 20 | 28649.396  (22132.484-36001.315) | 2588.925  (2018.796-3208.217) | 3 | 3.595  (3.083-4.109) |
| Liberia | 10395.251  (8581.526-12622.972) | 875.791  (728.498-1057.826) | 33 | 34737.855  (26822.430-44954.611) | 1403.379  (1106.106-1788.094) | 24 | 1.602  (1.483-1.721) |
| Madagascar | 47795.510  (39881.484-57324.912) | 899.935  (746.809-1077.332) | 32 | 123960.606  (97861.783-156874.615) | 995.296  (788.961-1257.695) | 44 | 0.313  (0.259-0.366) |
| Malawi | 42645.564  (36097.349-50178.454) | 1074.510  (915.652-1262.239) | 22 | 96853.285  (80414.863-115309.069) | 1217.262  (1013.698-1441.889) | 31 | 0.120  (-0.141-0.381) |
| Mali | 44849.951  (37818.252-53605.252) | 1075.278  (913.195-1281.627) | 21 | 161558.518  (130139.123-203330.809) | 1630.613  (1321.742-2026.381) | 13 | 1.438  (1.346-1.530) |
| Mauritania | 8263.448  (6839.764-9770.964) | 809.174  (667.455-955.774) | 35 | 22926.790  (18104.954-28642.975) | 1026.306  (807.306-1290.736) | 42 | 0.565  (0.489-0.642) |
| Mauritius | 13432.151  (12207.640-14970.509) | 1738.019  (1575.400-1932.088) | 5 | 62831.626  (56824.651-70751.953) | 3385.949  (3066.400-3810.035) | 1 | 3.315  (2.624-4.010) |
| Mozambique | 60800.973  (51865.379-71672.932) | 979.601  (847.152-1139.231) | 25 | 178462.070  (139084.912-219706.104) | 1419.143  (1118.986-1727.944) | 23 | 1.684  (1.511-1.858) |
| Namibia | 9579.035  (8130.158-11737.490) | 1470.109  (1247.080-1783.944) | 8 | 25760.064  (20244.697-32113.917) | 1866.292  (1485.357-2307.837) | 9 | 0.550  (0.243-0.858) |
| Niger | 20338.735  (16339.597-24823.568) | 673.718  (544.383-818.482) | 43 | 89355.097  (70168.777-114030.832) | 978.744  (772.517-1242.687) | 45 | 1.217  (1.140-1.294) |
| Nigeria | 409757.110  (346765.147-488700.106) | 908.687  (774.952-1073.445) | 29 | 1015687.227  (821899.323-1251125.167) | 1051.231  (869.329-1272.176) | 40 | 0.367  (0.317-0.418) |
| Rwanda | 43312.071  (31981.557-56441.906) | 1445.945  (1070.749-1888.839) | 9 | 69064.419  (46573.972-95241.169) | 1059.715  (720.832-1457.247) | 39 | -1.968  (-2.339--1.596) |
| Sao Tome and Principe | 329.342  (271.279-413.373) | 497.725  (411.471-622.200) | 46 | 1101.811  (874.690-1400.168) | 875.708  (705.587-1112.047) | 47 | 1.816  (1.737-1.895) |
| Senegal | 33159.040  (27797.198-40047.782) | 971.734  (814.811-1164.963) | 26 | 128365.217  (101895.559-158056.809) | 1561.188  (1234.294-1906.039) | 15 | 1.615  (1.480-1.750) |
| Seychelles | 382.305  (326.892-455.436) | 673.062  (575.238-801.513) | 44 | 1772.879  (1388.845-2263.835) | 1490.674  (1171.712-1891.494) | 19 | 2.730  (2.572-2.888) |
| Sierra Leone | 14998.400  (12471.051-18106.513) | 709.125  (592.368-856.650) | 39 | 47272.268  (38136.473-60232.154) | 1122.226  (910.864-1406.316) | 35 | 1.651  (1.465-1.837) |
| South Africa | 262094.390  (237877.454-291327.051) | 1220.260  (1105.990-1351.554) | 16 | 1010377.293  (914208.015-1116270.556) | 2154.163  (1956.601-2375.613) | 5 | 2.117  (1.740-2.495) |
| South Sudan | 29874.878  (22603.434-39280.349) | 1142.534  (868.716-1497.325) | 19 | 61854.986  (48011.940-81074.956) | 1481.390  (1155.353-1941.667) | 20 | 0.735  (0.574-0.897) |
| Togo | 9101.984  (7617.744-10995.504) | 678.27  3(562.691-818.445) | 42 | 43606.674  34382.382-55196.341) | 1050.290  (847.057-1312.792) | 41 | 1.386  (1.222-1.551) |
| Uganda | 65697.058  (47801.925-99424.676) | 1005.504  (733.065-1511.476) | 24 | 191264.366  (143891.080-265993.120) | 1210.502  (903.459-1651.515) | 32 | 0.136  (-0.095-0.368) |
| United Republic of Tanzania | 100613.761  (84716.747-117854.792) | 905.097  (770.385-1048.450) | 31 | 277039.318  (222197.634-345047.152) | 1023.642  (831.672-1266.440) | 43 | 0.226  (0.147-0.305) |
| Zambia | 38957.398  (33047.458-45695.199) | 1283.431  (1100.594-1511.937) | 15 | 114848.545  (91008.257-141630.335) | 1431.070  (1154.877-1746.342) | 22 | -0.085  (-0.281-0.112) |
| Zimbabwe | 39005.537  (32349.041-45747.391) | 956.970  (795.311-1118.613) | 27 | 133241.671  (107315.260-163900.363) | 1847.484  (1502.552-2244.450) | 10 | 2.695  (2.133-3.259) |
