# Supplementary figures and images for: African regional and national burden of diabetes mellitus and its attributable risk factors from 1990 to 2021: results from the global burden of disease study 2021
Source: Front Endocrinol (Lausanne). 2025 Aug 28;16:1643999. doi: 10.3389/fendo.2025.1643999 (PMC12422890; doi:10.3389/fendo.2025.1643999)

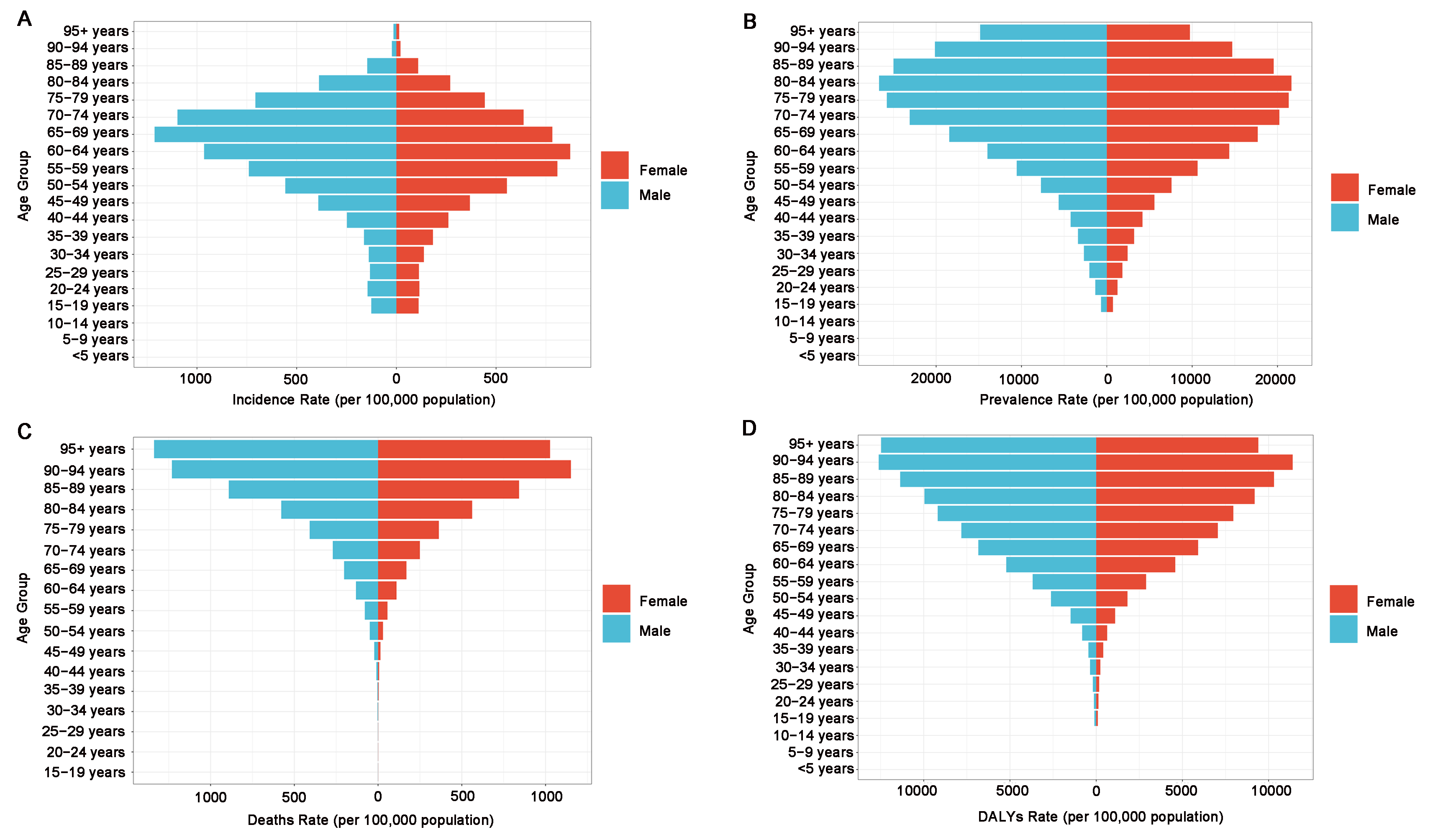

Supplement: Supplementary file 2 [file Image1.tif]

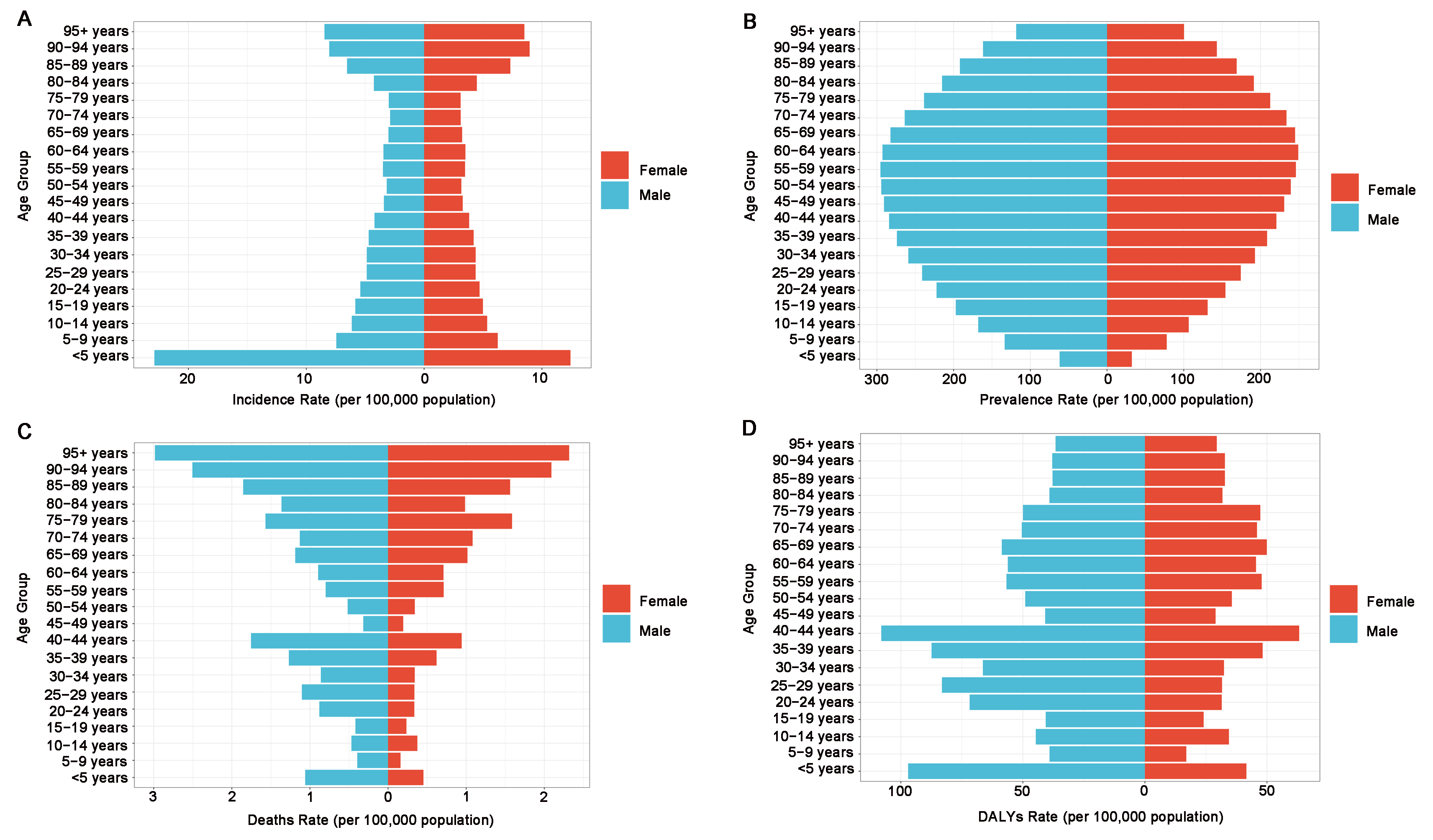

Supplement: Supplementary file 3 [file Image2.tif]

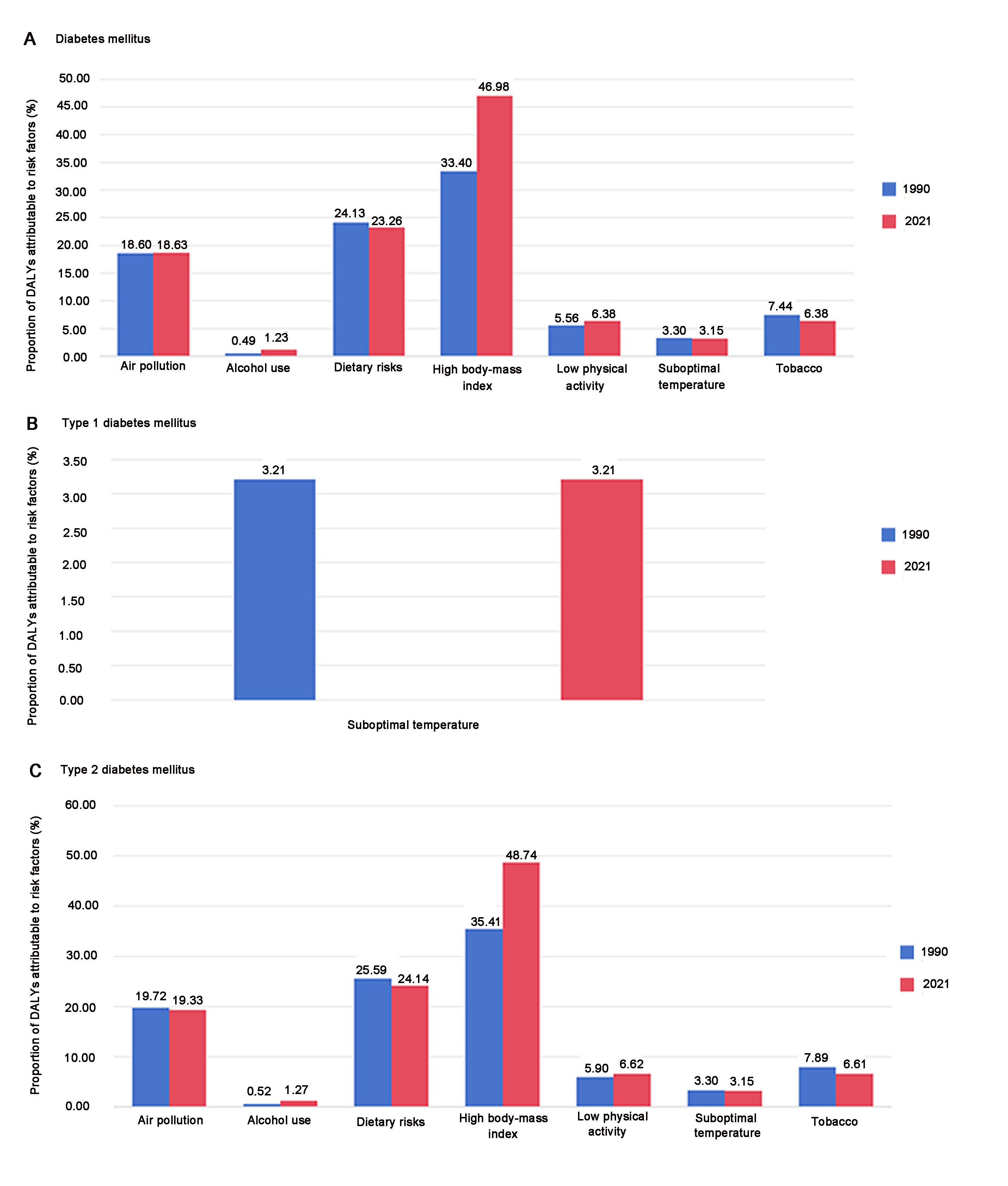

Supplement: Supplementary file 4 [file Image3.tif]
